# Sex and Individual Differences in Alcohol Intake Are Associated with Differences in Ketamine Self-Administration Behaviors and Nucleus Accumbens Dendritic Spine Density

**DOI:** 10.1523/ENEURO.0221-19.2019

**Published:** 2019-12-03

**Authors:** Caroline E. Strong, Katherine N. Wright, Mohamed Kabbaj

**Affiliations:** Program in Neuroscience, Department of Biomedical Sciences, Florida State University, Tallahassee, Florida 32306

**Keywords:** addiction, alcohol, individual differences, ketamine, rats, sex differences

## Abstract

Clinical and preclinical studies have shown that ketamine, an NMDA receptor antagonist, has promising therapeutic value for the treatment of alcohol use disorder (AUD). However, the maintenance of remission will ultimately require repeated infusions of ketamine, which may lead to abuse potential and may hinder its therapeutic benefits. It is therefore crucial to assess the effects of repeated treatments with ketamine on alcohol intake. Accordingly, this study aimed to examine in both sexes how individual differences in alcohol intake alter ketamine self-administration and how ketamine self-administration will alter subsequent alcohol-drinking behaviors. Male and female rats intermittently drank alcohol or water for 10 weeks and were divided into high- or low-alcohol intake groups prior to ketamine self-administration. Rats self-administered ketamine under fixed and progressive ratio schedules of reinforcement from week 4 to 7, and the incubation of ketamine craving was examined from week 8 to 10. To investigate structural plasticity in a brain region involved in reward, nucleus accumbens dendritic spine morphology was examined. Our results show that high alcohol intake in male rats attenuated ketamine self-administration, whereas in female rats high alcohol intake enhanced motivation to self-administer ketamine. Ketamine reduced alcohol intake in high-alcohol male rats but increased it in low-alcohol female rats. Incubation of ketamine craving developed in all groups except low-alcohol females. Three weeks of abstinence from ketamine was associated with increased mushroom spines in all groups except the high-alcohol male group. Overall, these data suggest that ketamine as a treatment for AUD may benefit male subjects, but not female subjects, and warrants further investigation before use as a therapeutic agent.

## Significance Statement

Alcohol use disorder (AUD) is one of the most prevalent forms of addiction, yet effective treatment options are lacking. Preclinical and clinical data suggest that ketamine is a potential AUD treatment option. Since ketamine is also an addictive drug, we investigated here the relationship between alcohol and ketamine in both sexes and examined morphologic changes in nucleus accumbens (NAc) medium spiny neurons, which are involved in mediating addiction-related plasticity. We showed clear individual and sex differences in the interactions between alcohol and ketamine, which were reflected with structural plasticity alterations in the NAc of both sexes. Our data suggest that ketamine could be further investigated in humans as a viable treatment for AUD in male subjects, but not in female subjects.

## Introduction

Alcohol use disorder (AUD) is a chronic relapsing disorder characterized by compulsive alcohol (Alc) use despite negative consequences (American Psychiatric Association, 2013). While most alcohol consumers are able to control their intake, 15% of the population worldwide is susceptible to pathologic drinking patterns symptomatic of AUD (Substance Abuse and Mental Health Services Administration, 2015). Within this subset, gender differences in alcohol intake and relapse exist, suggesting a need for further examination into AUD treatment options within both sexes, particularly because 75% of people experiencing AUD relapse within 1 year (Miller et al., 2001; Becker and Koob, 2016). As such, there is a critical need to gain a better understanding of the neuroadaptations associated with alcohol addiction within both sexes to improve treatment options and prevent relapse.

Ketamine (Ket), a NMDA receptor antagonist, has shown promise as a potential AUD treatment option, demonstrated by preclinical and clinical studies showing that acute, low-dose administration can reduce alcohol intake in chronically drinking rats and humans (Sabino et al., 2013b; Holleran et al., 2016; Rezvani et al., 2017; ClinicalTrials.gov, Identifier: NCT03658330). However, as a schedule III drug, ketamine itself is addictive and has great potential for abuse and dependence in humans (Narendran et al., 2005). Furthermore, preclinical studies suggest that male rats will self-administer ketamine across a wide range of doses, and recently these findings were extended to rats of both sexes, showing that female rats are more sensitive to the addictive effects of ketamine than male rats (De Luca and Badiani, 2011; Venniro et al., 2015; Strong et al., 2017; Schoepfer et al., 2019; Wright et al., 2019).

To date, studies examining ketamine as an AUD treatment option have only investigated acute ketamine administration despite the fact that in clinical settings, alcoholic patients would require repeated ketamine infusions to maintain sobriety. This is a major safety concern since ketamine, when used chronically, can alter plasticity in reward-related brain regions such as the nucleus accumbens (NAc), which is considered the hub of reward circuitry and is heavily involved in drug-seeking behavior (Mogenson et al., 1980; Heimer et al., 1997; Groenewegen et al., 1999). In the NAc, such plasticity can occur through morphologic alterations in medium spiny neurons (MSNs) via dynamic changes in dendritic spines, which are indicative of AMPA receptor trafficking to the synapse, thus increasing cell excitability and inducing synaptic plasticity (Russo et al., 2010). In the NAc, repeated ketamine exposure increases dendritic spine density and protein expression of AMPA and NMDA glutamate receptors in the NAc, suggesting enhanced glutamatergic transmission (Caffino et al., 2017; Strong et al., 2017; Yao et al., 2018). However, it is important to keep in mind that the effects of ketamine are widespread, and it can act on a variety of neurotransmitter systems, including but not limited to glutamatergic, dopaminergic, and serotonergic pathways (Can et al., 2016; Pham et al., 2017; Rincón-Cortés and Grace, 2017).

Similarly, chronic exposure to alcohol enhances NMDA-mediated plasticity within the NAc (Dildy and Leslie, 1989; Roberto and Varodayan, 2017). Chronic alcohol intake also increases NAc dendritic spine density and potentiation of AMPA and NMDA function (Uys et al., 2016; Ji et al., 2017; Laguesse et al., 2017; Kircher et al., 2019). As such, inhibiting glutamate receptors may be a key component in the treatment of AUD. Thus, while acute ketamine treatment may be beneficial for AUD treatment, it seems necessary, for therapeutic reasons, to further delve into how ketamine under a repeated regimen will affect alcohol drinking.

Therefore, the overall aim of this study was to investigate the relationship between alcohol and ketamine under repeated exposure paradigms. To better model AUD in humans, we investigated individual differences in alcohol intake and examined how each of these subgroups self-administered Ket or saline (Sal). We then assessed how ketamine or saline self-administration (SA) impacted alcohol intake. Finally, we examined dendritic spine density in the NAc to investigate how alcohol and ketamine, in combination, affected NAc structural plasticity in both sexes.

## Materials and Methods

### Animals

Male Sprague Dawley rats weighing 225–250 g and female Sprague Dawley rats weighing 175–200 g were obtained from Charles River (total, *n* = 52 males, 52 females; 8 weeks of age). We maintained rats on a reverse 12 h light/dark cycle (lights on at 10:00 P.M., off at 10:00 A.M.) with food and water available *ad libitum*, except during experimental testing. Same-sex rats were pair housed in 43 × 21.5 × 25.5 cm Plexiglas cages before the start of alcohol intake, but were singly housed on days where rats consumed alcohol (Monday, Wednesday, and Friday). Once the alcohol paradigm began, cage mates were removed from their home cage and placed in isolated housing conditions within the same room during alcohol-intake sessions. Isolated cages contained environmental enrichment to reduce the effects of social isolation stress. After each 24 h alcohol session, cage mates were placed back into their home cage on alcohol deprivation days. All animal procedures were performed in accordance with the regulations of the Florida State University animal care committee.

### Drugs

Alcohol (Koptec) was prepared by diluting ethyl alcohol solution in deionized water to 20% (40 proof) v/v from a 200 proof solution. Racemic ketamine hydrochloride (Ketasthesia, Henry Schein Medical) was diluted in 0.9% sterile saline from a 100 mg/ml stock solution. For self-administration experiments, ketamine was administered intravenously at a rate of 0.5 mg/kg/infusion in a 50 μl volume.

### Surgical procedures

#### Intravenous catheterization surgery

Rats were anesthetized with isoflurane (Henry Schein Medical) at a rate of 4% for induction and 2% for maintenance at an oxygen flow rate of 1 L/min. Surgery was conducted as previously described (Wright et al., 2017), with minor modifications. Vascular access buttons were connected to indwelling intravenous SILASTIC catheters (Instech Laboratories), which were inserted into the right jugular vein. Catheters were flushed twice daily with 0.1 ml heparinized saline (50 U/ml; Alfa Aesar) and ampicillin (30 mg/ml; VWR). Rats were given a 3 d recovery period before operant self-administration testing. Catheter patency was tested by intravenously administering 0.05 ml of xylazine (2.5 mg/ml) on days where rats were not exposed to alcohol or ketamine (Sunday) each week during the self-administration period. If rapid loss of muscle tone was not observed, the catheter was no longer considered patent, and a new catheter was inserted into the left jugular vein. If a rat failed a second patency test, the rat was removed from the study and not included in statistical analyses. We excluded some rats because of the failure of catheter patency (*n* = 2) during operant self-administration. The two excluded rats included one high-alcohol intake male that self-administered saline and one high-alcohol intake female that self-administered saline.

#### Intracranial viral construct delivery

Twenty-four hours after the incubation of ketamine craving test on day 21, we bilaterally injected 1 μl (1 × 10^9^ U/ml) of the viral construct HSV-CMV-GFP (Viral Core, McGovern Institute for Brain Research, Massachusetts Institute of Technology, Cambridge, MA) into the NAc over 5 min at a rate of 0.1 μl every 30 s, under the anesthetic conditions mentioned above. After 5 min of virus diffusion, the needles were raised, craniotomies were sealed with bone wax, and the incision was closed. The coordinates for the NAc were as follows (from the skull surface: anteroposterior, +1.5 mm; mediolateral, ±1.2 mm; dorsoventral, −7.6 mm). Rats were given 3 d to rest and allow for the optimal expression of HSV-CMV-GFP (Lachmann, 2004).

### Behavioral testing

#### Novelty response

All rats underwent an initial 1 h novelty-induced locomotor test before experimental testing, as previously described (Kabbaj, 2006). During the first 4 h of the dark cycle, rats were placed in circular chambers 71.2 cm in diameter (Med Associates) with four equidistant photobeam sensors that record locomotor movements based off number of beam breaks. This test allows the categorization of rats into high or low responders based on whether locomotor scores are above or below the median score. This test was not used as an independent variable in any of the analyses but was taken into consideration when assigning and balancing experimental groups.

#### Alcohol intake: high versus low drinkers

The intermittent access to 20% alcohol two-bottle choice (IA2BC20%) paradigm was used in the current study to model alcohol intake in rodents (Carnicella et al., 2014). Rats were given 24 h of concurrent access to one bottle of 20% alcohol and one bottle of water while control rats were given access to two bottles of water. Drinking sessions started at the onset of the dark cycle (10:00 A.M.) and occurred 3 d/week (Monday, Wednesday, and Friday) for 10 weeks. The side placement of the alcohol bottle (right or left) was alternated for each session to avoid development of a side preference. On alcohol deprivation days, rats were exposed to two bottles of water. To calculate the amount of fluid consumed from the bottle containing alcohol, the bottle was weighed before and after each drinking session. The difference in weights was used as the amount of alcohol consumed in milliliters. Rats were weighed at the beginning of each alcohol session to calculate the dose of alcohol consumed per session, and the following equation was used to calculate the dose of alcohol consumed (in grams per kilogram) within the 24 h period: (alcohol intake (ml) * 0.2)/(body weight in kilograms). The percentage preference for alcohol was calculated as follows: [alcohol consumed (ml)/total fluid consumed (ml)] * 100.

To assess the role that individual differences in alcohol consumption would have on ketamine self-administration, rats were divided into high-alcohol intake and low-alcohol intake subgroups. Because female rats drank significantly more alcohol than male rats, a frequency distribution within each sex was used to assess whether average alcohol consumption was normally distributed, and a median split was used to divide rats based on high-alcohol (*n* = 15 females, 15 males) and low-alcohol (*n* = 16 females, 16 males) intake. Average alcohol consumption data during the third week were used so that groups could be established before ketamine self-administration.

#### Operant training

Before initiation of the IA2BC20% paradigm, animals were trained under a fixed ratio 1 (FR1) schedule of reinforcement for 1 h each day to respond to 45 mg sucrose pellets, as previously described (Wright et al., 2017) but with minor modifications. Self-administration chambers (30.5 × 24.1 × 21 cm; Med Associates) within sound-attenuating cabinets contained two nose-poke holes and an automated food hopper on the opposite wall. An active response resulted in the delivery of one sucrose pellet with the house light turning off and the cue light above the nose-poke hole turning on for 20 s. Within this 20 s time-out period, active responses were still recorded but did not result in a sucrose reward. Rats trained for 5 d to meet the criteria of ≤30% variability between sessions and ≥70% more active than inactive responses within each session.

#### Ketamine self-administration and incubation of craving

Intravenous self-administration of either ketamine or saline began during week 4 of the IA2BC20% paradigm. Given that alcohol sessions only occurred 3 d/week (Monday, Wednesday, and Friday), ketamine self-administration sessions occurred on days when rats did not have exposure to alcohol (Tuesday, Thursday, and Saturday) at least 2 h after the removal of alcohol bottles. Rats intermittently (3 d/week) self-administered ketamine or saline in the same operant chambers that sucrose pellet training occurred over a 4 week period, for a total of 11 sessions. Sessions 1–6 and sessions 10–11 were 2 h sessions under an FR1 schedule of reinforcement with 100 maximum infusions. The active nose poke resulted in one infusion and triggered drug-paired cues that were identical to the sucrose pellet training sessions, while the inactive nose poke resulted in no programmed response. Each infusion resulted in intravenous delivery of 50 μl of ketamine or saline through polyethylene tubing protected by a metal spring and attached via swivel to tubing (Instech) connected to a 10 ml syringe outside of the chamber. The three progressive ratio (PR) sessions 7–9 nested between FR1 session 6 and 10, where the number of active responses for one infusion increased exponentially (1, 2, 4, 6, 9, 12, 15, 20, 32…) according to the equation 5 * e^(0.2n)−5^, where *n* equals the infusion number (Richardson and Roberts, 1996). PR sessions ended if the rat did not achieve the next ratio within 1 h. Rats were then retrained on FR1 for sessions 10 and 11 before the examination of incubation of ketamine craving on days 1, 7, and 21 after the last ketamine self-administration. After the last FR1 session (session 11), rats underwent a 21 d forced abstinence period within their home cage. During this period, rats were re-exposed to the operant chamber for three sessions occurring on days 1, 7, and 21 to assess the incubation of ketamine craving, as described in previous studies (Lu et al., 2004; Pickens et al., 2011; Venniro et al., 2016). The incubation of craving tests was identical to the FR1 sessions during the self-administration period, except drug infusions did not occur after each active response. Twenty-four hours after the final incubation test, HSV-CMV-GFP was bilaterally injected into the NAc of a subset of rats within each treatment group (*n* = 3–4/group), as described above, and rats were killed 3 d later.

#### Immunohistochemistry

Three days after HSV-CMV-GFP was delivered to the NAc, rats were anesthetized with sodium pentobarbital (Socumb, Henry Schein Medical) and transcardially perfused with 0.2 m PBS and 4% paraformaldehyde (PFA) in PBS. Brains were extracted and postfixed in PFA for 24 h, then were transferred to 0.2 m PBS + 0.01% sodium azide at 4°C. Brains were sectioned on a vibratome (model VT1200S, Leica) at 150 μm thickness. Sections containing NAc underwent three PBS washes followed by a 1 h incubation period in 0.4% Triton X-100 in PBS with gentle agitation. Tissue was blocked for 1 h in a PBS solution containing 5% normal goat serum, 5% bovine serum solution, and 0.4% Triton X-100. Tissue was placed on a rocker overnight at 4°C in primary antibody (1:500; anti-GFP, chicken, polyclonal, Abcam) in blocking buffer. After a series of washes in PBS + 0.4% Triton X-100, sections were placed in secondary antibody (1:1000; Alexa Fluor 488, goat anti-chicken, Thermo Fisher Scientific) on a rocker overnight at 4°C. Sections were slide mounted on Fisher SuperFrost slides (Thermo Fisher Scientific) the following day and coverslipped after the application of Vectashield mounting medium (Vector Laboratories).

#### Image acquisition and dendritic spine quantification

Images of dendritic spines in the NAc were acquired on a laser-scanning confocal microscope (LSM 780, Zeiss) at a 63× objective with a 1.4 oil-immersion numerical aperture. Sixteen bit *z*-stacks were collected at 2× Nyquist with a 0.2 μm *z*-step during excitation with the 488 laser. Dendritic segments were imaged if they were >50 μm from the soma, past the first branching point, and 40–55 μm in length. In treatment groups with four rats/group, at least 8 dendritic segments per rat were imaged; and in groups with three rats/group, 10–12 segments/rat were imaged. Two rats were removed from the analysis due to lack of GFP expression within the NAc. Compressed image stacks were deconvolved using AutoQuantX, and semi-automated 3D reconstruction of dendritic spines was conducted in Neurolucida 360. Briefly, dendritic spine segments >50 μm from the soma were traced with the user-guided setting ranging in length from 40 to 55 μm. The automatic dendritic spine identification and classification from Neurolucida 360 was used to reconstruct spines and classify spine subtypes. For quantification, reconstructed dendritic segments were uploaded to Neurolucida Explorer to assess the branch segment length, and number of total, thin, mushroom, and stubby spines.

### Experimental design

**Figure 1.**
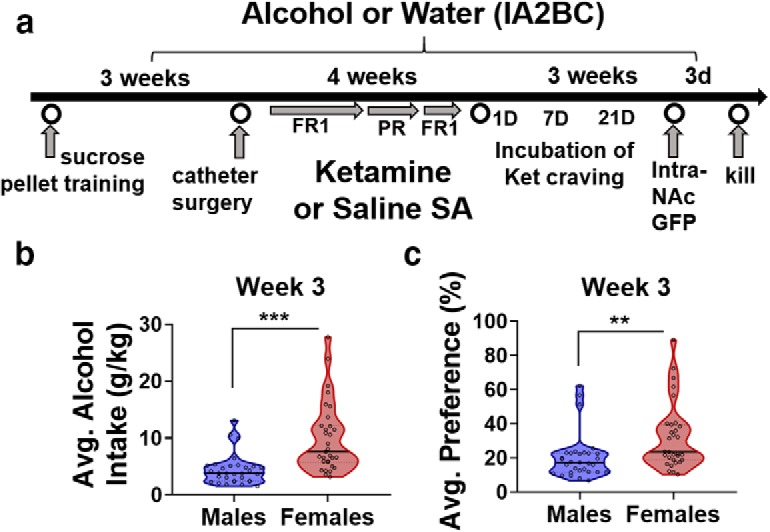
Establishing high versus low alcohol intake in male and female rats. ***a***, Time line of experiment: rats underwent 10 weeks of intermittent access to the IA2BC20% and water paradigm, with ketamine self-administration nested from week 4 to 7 and incubation of ketamine-craving tests from week 8 to 10. Rats received bilateral HSV-GFP injections into the NAc after 10 weeks of alcohol intake and were killed 3 d later. ***b***, ***c***, Distribution of alcohol intake (in grams per kilogram) and preference (percentage) for alcohol during the third week of intake. A median split was used to determine cutoffs for high and low alcohol intake: high-alcohol intake male rats (*n* = 15), low-alcohol intake male rats (*n* = 16); high-alcohol intake female rats (*n* = 15), and low-alcohol intake female rats (*n* = 16). **p < 0.01, ***p < 0.001. Data are represented as mean ± SEM average alcohol intake (***a***) and preference (***b***).

The goal of the current study was to assess whether a repeated regimen of ketamine self-administration will affect alcohol drinking and, in turn, how alcohol drinking will affect ketamine self-administration in male and female rats. To do so, rats were subjected to the IA2BC20% alcohol paradigm for 10 weeks, with ketamine self-administration nested from week 4 to 7 (Fig. 1*a*, time line). Rats were subjected to sucrose pellet training before being exposed to alcohol or ketamine. The design of the study was nonrandom in that groups were assigned based on intake (low or high alcohol intake). Intravenous self-administration (ketamine vs saline) was assigned after 3 weeks of alcohol or water intake, and was based on locomotor responses to novelty, sucrose pellet training, alcohol intake (if applicable), and body weight. Ketamine self-administration began 24 h after session 9 (week 3) of alcohol intake. From week 4 to 7, rats were subjected to ketamine or saline self-administration on days when they were not drinking alcohol (Alc: Monday, Wednesday, and Friday; Ket: Tuesday, Thursday, and Saturday). The 4 week ketamine self-administration period consisted of six FR1 sessions followed by three PR and two more FR1 sessions to assess the acquisition of drug taking, the motivation to self-administer, and restabilization of the rats on their initial schedule of reinforcement, respectively. Twenty-four hours, 7 d, and 21 d after the last ketamine session, the incubation of ketamine craving was tested where rats were re-exposed to the operant chambers and drug-related cues but received no drug reward. Twenty-four hours after the incubation of craving test on day 21, HSV-CMV-GFP was bilaterally injected into the NAc. During the 3 d recovery period to allow peak viral expression, rats were exposed to one final alcohol or water session and transcardially perfused 24 h later. Once brain tissue sections containing the NAc were sectioned at 150 μm, immunohistochemistry using the primary antibody anti-GFP was performed. NAc sections were slide mounted and coverslipped before imaging on a laser-scanning confocal microscope. 3D images were reconstructed and quantified using Neurolucida 360 and Neurolucida Explorer, respectively.

### Statistical analysis

See Table 1 for a detailed description of statistical analyses conducted throughout the experiment. Behavioral and morphologic data were analyzed with RStudio (version 3.5.1) using a linear mixed-model framework for ANOVAs, while correlations were analyzed within GraphPad Prism. All behavioral experiments were analyzed using four-way linear mixed-model ANOVAs where sex, intake, and treatment were the between-subjects factor and session was the within-subjects factor. Given that time was treated as linear, we analyzed the effect of sessions using repeated measures within a mixed-model framework. Spine density data were analyzed with three-way linear mixed-model ANOVA with sex, intake, and treatment as between-subjects factors. To assess the effects of intake, treatment, and sessions within each sex, we followed up ANOVAs with a significant main effect of sex with two-way (spine density analyses) or three-way (behavior analyses) linear mixed-model ANOVAs within males and females. The packages nlme (Pinheiro et al., 2018), lme4 (Bates et al., 2015), and lsmeans (Lenth, 2016) were used for all statistical analyses and figures. Significant interactions or significant main effects (α = 0.05) were followed by Tukey’s *post hoc* test. Figures were created using Prism (version 8.01, GraphPad)

**Table 1:** Detailed statistical table

	Figure	Comparison	Type of test	Statistic	95% CI
a	NA	Sucrose pellets	Four-way LMM ANOVA	Sex × intake × treatment × sessions: *F*_(8,364)_ = 0.85	*p* = 0.56
b	NA	Alcohol (g/kg), weeks 1–3	Three-way LMM ANOVA	Sex: *F*_(1,59)_ = 48.42	*p* < 0.0001
c	NA	Alcohol (%pref), weeks 1–3	Three-way LMM ANOVA	Sex: *F*_(1,58)_ = 17.61	*p* < 0.0001
d	1*b,c*	Week 3 average alcohol intake and preference	Unpaired *t* tests	Intake: *t*_(60)_ = 4.66 Preference: *t*_(60)_ = 2.74	*p* < 0.0001 *p* = 0.008
	1*b*	Average alcohol (g/kg)	Frequency distribution, median split	Cutoff males: 4.3 g/kg females: 6.9 g/kg	NA
	1*c*	Average alcohol (%)	Frequency distribution, median split	Cutoff males: 16%, females: 23%	NA
e	NA	Body weight, weeks 1–3	Three-way LMM ANOVA	Sex: *F*_(1,93)_ = 736.83	*p* < 2e^−16^
	NA	Body weight, weeks 1–10	Four-way LMM ANOVA	Sex × intake × treatment × sessions: *F*_(60,2790)_ = 1.57	*p* = 0.004
	NA	Males: body weight, weeks 1–10	Three-way LMM ANOVA	Intake × treatment × sessions: *F*_(60,1410)_ = 1.75	*p* = 0.0004
	NA	Water intake: Ket SA vs Sal SA	Tukey’s *post hoc*	Sessions 17–31: *t* < −2	Sessions 17-31: *p* < 0.05
g	2*a,b*	FR1 infusions, sessions 1–6	Four-way LMM ANOVA	Sex: *F*_(1,93)_ = 9.02	*p* = 0.0034
	2*a,b*	FR1 Infusions, sessions 10–11	Four-way LMM ANOVA	Sex × intake: *F*_(2,93)_ = 3.45	*p* = 0.036
	2*a,b*	High-Alc: F vs M	Tukey’s *post hoc*	SA sessions 10–11: *t*_(93)_ = 3.05	*p* = 0.003
	2*a,b*	Males: FR1 Infusions, sessions 1–6	Three-way LMM ANOVA	Intake × treatment × sessions: *F*_(10,230)_ = 2.3	*p* = 0.014
	2*a*	Males_Ket SA: high vs low and high vs water	Tukey’s *post hoc*	SA session 4: *t*_(46)_ = −2.8 SA session 5: *t*_(46)_ = −3.13	Session 4: *p* = 0.02 Session 5: *p* =0.008
		Males: FR1 infusions, sessions 10–11	Three-way LMM ANOVA	Intake × treatment: *F*_(2,46)_ = 3.83	*p* = 0.029
		Ket SA: high-Alc vs water	Tukey’s *post hoc*	*t*_(46)_ = −2.5	*p* = 0.04
		Ket SA: high-Alc vs low_Alc	Tukey’s *post hoc*	*t*_(46)_ = −3.005	*p* = 0.012
	2*a,b*	Females: FR1 Infusions, sessions 1–6	Three-way LMM ANOVA	Treatment × sessions: *F*_(5,235)_ = 38.03	*p* < 0.0001
		Females: FR1 Infusions, sessions 10–11	Three-way LMM ANOVA	Treatment × sessions: *F*_(1,47)_ = 6.11	*p* = 0.017
h	2*c,d*	Active responses, sessions 1–6	Four-way LMM ANOVA	Sex × intake × treatment × sessions: *F*_(10,459)_ = 2.13	*p* = 0.021
	2*c*	Ket SA_water: F vs M	Tukey’s *post hoc*	SA session 2: *t*_(93)_ = 2.33	*p* = 0.022
	2*c*	Ket SA_low-Alc: F vs M	Tukey’s *post hoc*	SA sessions 5–6: *t*_(93)_ = 2.18, 2.62	*p* = 0.032, 0.01
	2*c*	Ket SA_high-Alc: F vs M	Tukey’s *post hoc*	SA sessions 3–6: *t*_(93)_ = 3.51, 3.53, 4.54, 4.59	*p* = 0.0007, 0.0007, 0.0001, 0.0001
	2*c,d*	Active responses, sessions 10–11	Four-way LMM ANOVA	Sex: *F*_(1,93)_ = 9.93	*p* = 0.002
	2*c,d*	Inactive responses, sessions 1–6	Four-way LMM ANOVA	Sex: *F*_(1,94)_ = 4.1	*p* = 0.04
		Inactive responses, sessions 10–11	Four-way LMM ANOVA	Sex × intake × treatment × sessions: *F*_(2,90)_ = 0.35	*p* = 0.71
	2*c,d*	Males: active responses, sessions 1–6	Three-way LMM ANOVA	Intake × treatment × sessions: *F*_(10,228)_ = 3.13	*p* = 0.0009
	2*c*	Males_Ket SA: high vs water	Tukey’s *post hoc*	SA sessions 5–6: *t*_(46)_ = −3.59, −2.95	*p* = 0.002, 0.01
	2*c,d*	Males: active response, sessions 10–11	Three-way LMM ANOVA	Intake × treatment: *F*_(2,46)_ = 3.65	*p* = 0.034
	2*c*	Males_Ket SA: high vs water	Tukey’s *post hoc*	Main effect of intake: *t*_(46)_ = −3.17	*p* = 008
	2*c,d*	Females: active response, sessions 1–6	Three-way LMM ANOVA	Treatment × sessions: *F*_(5,231)_ = 26.44	*p* < 0.0001
	2*c,d*	Females: active response, sessions 10–11	Three-way LMM ANOVA	Intake × treatment × sessions: *F*_(2,46)_ = 4.12	*p* = 0.02
	2*c*	Ket SA: high-Alc vs water	Tukey’s *post hoc*	SA session 10: *t*_(46)_ = 2.77	*p* = 022
i	3*a,b*	PR break point	Four-way LMM ANOVA	Sex × intake × treatment × sessions: *F*_(4,186)_ = 3.6	*p* = 0.0074
	3*a*	Ket SA_water: F vs M	Tukey’s *post hoc*	SA session 7: *t*_(93)_ = 2.21	*p* = 0.029
	3*a*	Ket SA_low-Alc: F vs M	Tukey’s *post hoc*	SA session 9: *t*_(93)_ = 3.29	*p* = 0.0014
	3*a*	Ket SA_high-Alc: F vs M	Tukey’s *post hoc*	SA sessions 8–9: *t*_(93)_ = 5.27, 3.32	*p* = 0.0001, 0.0013
	3*a,b*	Males: break point	Three-way LMM ANOVA	Treatment × sessions: *F*_(2,92)_ = 3.21	*p* = 0.045
	3*a,b*	M_Ket vs Sal	Tukey’s *post hoc*	SA sessions 7–8: *t*_(46)_ = 4.08, 2.94	*p* = 0.0002, 0.0052
	3*a,b*	Females: break point	Three-way LMM ANOVA	Intake × treatment × sessions: *F*_(4,94)_ = 3.67	*p* = 0.008
	3*a,b*	F_Water: Ket vs Sal SA	Tukey’s *post hoc*	SA session 7: *t*_(47)_ = 2.25	*p* = 0.029
	3*a,b*	F_Low-Alc: Ket vs Sal SA	Tukey’s *post hoc*	SA session 9: *t*_(47)_ = 2.52	*p* = 0.015
	3*a,b*	F_High-Alc: Ket vs Sal SA	Tukey’s *post hoc*	SA session 8: *t*_(47)_ = 3.67	*p* = 0.0006
	3*a*	F_Ket SA: high vs water and high vs low	Tukey’s *post hoc*	SA session 8: *t*_(47)_ = 3.94, 3.78	*p* = 0.0008, 0.012
j	3*c,d*	PR active responses	Four-way LMM ANOVA	Sex × intake × treatment × sessions: *F*_(4,185)_ = 3.25	*p* = 0.013
	3*c*	Ket SA_water: F vs M	Tukey’s *post hoc*	SA session 7: *t*_(93)_ = 1.99	*p* = 0.049
	3*c*	Ket SA_low-Alc: F vs M	Tukey’s *post hoc*	SA session 9: *t*_(93)_ = 3.27	*p* = 0.0015
	3*c*	Ket SA_high-Alc: F vs M	Tukey’s *post hoc*	SA sessions 8–9: *t*_(93)_ = 5.2, 3.43	*p* = 0.0001, 0.0009
	3*c,d*	Males: PR active responses	Three-way LMM ANOVA	main effect of treatment: *F*_(1,46)_ = 10.43	*p* = 0.0023
	3*c,d*	Females: active responses	Three-way LMM ANOVA	Intake × treatment × sessions: *F*_(4,94)_ = 3.25	*p* = 0.015
	3*c,d*	F_low-Alc: Ket vs Sal SA	Tukey’s *post hoc*	SA session 9: *t*_(47)_ = 2.48	*p* = 0.017
	3*c,d*	F_high-Alc: Ket vs Sal SA	Tukey’s *post hoc*	SA session 8: *t*_(47)_ = 3.65, 2.05	*p* = 0.0007, 0.0047
	3*c*	F_Ket SA: high vs water and high vs low	Tukey’s *post hoc*	SA session 8: *t*_(47)_ = 3.88, 3.76	*p* = 0.0009, 0.0014
k	3*e,f*	PR infusions	Four-way LMM ANOVA	Sex × intake × treatment × sessions: *F*_(4,186)_ = 2.95	*p* = 0.022
	3*e*	Ket SA_water: F vs M	Tukey’s *post hoc*	SA session 7: *t*_(93)_ = 2.26	*p* = 0.026
	3*e*	Ket SA_low-Alc: F vs M	Tukey’s *post hoc*	SA session 9: *t*_(93)_ = 2.69	*p* = 0.0084
	3*e*	Ket SA_high-Alc: F vs M	Tukey’s *post hoc*	SA sessions 8–9: *t*_(93)_ = 4.04, 3.54	*p* = 0.0001, 0.0006
	3*e,f*	Males: infusions	Three-way LMM ANOVA	Treatment × sessions: *F*_(2,92)_ = 4.32	*p* = 0.016
	3*e,f*	M_Ket vs Sal	Tukey’s *post hoc*	SA sessions 7–9: *t*_(46)_ = 5.48, 4.17, 2.09	*p* = 0.0001, 0.0001, 0.04
	3*e,f*	Females: infusions	Three-way LMM ANOVA	Intake × treatment × sessions: *F*_(4,94)_ = 4.48	*p* = 0.002

	3*e,f*	F_water: Ket vs Sal SA	Tukey’s *post hoc*	SA sessions 7, 9: *t*_(47)_ = 3.82, 2.03	*p* = 0.0004, 0.049
	3*e,f*	F_low-Alc: Ket vs Sal SA	Tukey’s *post hoc*	SA session 9: *t*_(47)_ = 2.71	*p* = 0.005
	3*e,f*	F_high-Alc: Ket vs Sal SA	Tukey’s *post hoc*	SA session 8: *t*_(47)_ = 2.96	*p* = 0.009
	3*e*	F_Ket SA: high vs water and high vs low	Tukey’s *post hoc*	SA session 8: *t*_(47)_ = 2.9, 3.19	*p* = 0.02, 0.007
l	4*a,b*	Incubation of craving, active responses	Four-way LMM ANOVA	Sex: *F*_(1,85)_ = 9.81	*p* = 0.0024
	4*a,b*	Males: incubation of craving, active responses	Three-way LMM ANOVA	Treatment × sessions: *F*_(1,85)_ = 3.79	*p* = 0.026
	4*a,b*	Males: incubation of craving, active responses	Three-way LMM ANOVA	Intake × treatment: *F*_(2,44)_ = 5.44	*p* = 0.008
	4*a*	M_water, Ket SA: day 1 vs 21	Tukey’s *post hoc*	*t*_(91)_ = 2.52	*p* = 0.03
	4*a*	M_low-Alc, Ket SA: day 1 vs 7, 1 vs 21	Tukey’s *post hoc*	*t*_(91)_ = 3.44, 4.05	*p* = 0.0025, 0.0003
	4*a*	M_high-Alc, Ket SA, day 1 vs 7	Tukey’s *post hoc*	*t*_(91)_ = 2.72	*p* = 0.02
	4*a,b*	Females: incubation of craving, active responses	Three-way LMM ANOVA	Intake × treatment: *F*_(2,41)_ = 3.93	*p* = 0.027
	4*b*	F_water, Sal SA: day 1 vs 7, 1 vs 21	Tukey’s *post hoc*	*t*_(82)_ = 2.8, 2.8	*p* = 0.01, 0.01
	4*a*	F_water, Ket SA: day 1 vs 21	Tukey’s *post hoc*	*t*_(82)_ = 2.8	*p* = 0.01
	4*a*	F_high-Alc, Ket SA, day 1 vs 7	Tukey’s *post hoc*	*t*_(82)_ = 2.52	*p* = 0.04
m	5*a,b*	Alcohol (g/kg), weeks 1–10	Four-way LMM ANOVA	Sex: *F*_(1,53)_ = 83.87	*p* < 0.0001
	5*a,b*	Males: g/kg weeks 1–10	Three-way LMM ANOVA	Intake × treatment × sessions: *F*_(30,785)_ = 2.53	*p* < 0.0001
	5*a*	High-Alc: Ket SA vs Sal SA	Tukey’s *post hoc*	Sessions 19, 22: *t*_(27)_ < −2.68, −3.08	*p* = 0.019, 0.009
	5*a,b*	Females: g/kg weeks 1–10	Three-way LMM ANOVA	Intake × sessions: *F*_(30,760)_ = 2.5; treatment × sessions: *F*_(30,760)_ = 1.6	*p* < 0.0001; *p* = 0.022
	5*b*	Low-Alc: Ket SA vs Sal SA	Tukey’s *post hoc*	Sessions 19–20, 23–26: *t*_(27)_ > 2.26	*p* < 0.05
n	6*a,b*	Alcohol (%pref), weeks 1–10	Four-way LMM ANOVA	Sex: *F*_(1,53)_ = 6.19	*p* = 0.016
	6*a,b*	Males: %pref weeks 1–10	Three-way LMM ANOVA	Intake × treatment × sessions: *F*_(30,784)_ = 2.28	*p* = 0.0001
	6*a*	High-Alc: Ket SA vs Sal SA	Tukey’s *post hoc*	Sessions 19–23, 25–31: *t*_(27)_ < −2.07	p < 0.05
	6*a,b*	Females: %pref weeks 1–10	Three-way LMM ANOVA	Intake × treatment × sessions: *F*_(30,759)_ = 1.67	*p* = 0.015
	6*b*	Low-Alc: Ket SA vs Sal SA	Tukey’s *post hoc*	Sessions 19–20, 22–26: *t*_(26)_ > 2.11	*p* < 0.05
o	7*c*	Total spines	Three-way LMM ANOVA	Sex × intake: *F*_(2,26)_ = 4.77	*p* = 0.017
	7*c*	High-Alc: M vs F	Tukey’s *post hoc*	*t*_(26)_ = −4.17	*p* = 0.0003
	7*c*	Males: total spines	Two-way LMM ANOVA	Intake: *F*_(2,13)_ = 3.84	*p* = 0.04
		High-Alc vs water	Tukey’s *post hoc*	*t*_(15)_ = 2.87	*p* = 0.02
	7*c*	Females: total spines	Two-way LMM ANOVA	Intake: *F*_(2,13)_ = 16.23	*p* = 0.00029
p	7*d*	Thin spines	Three-way LMM ANOVA	Sex: *F*_(1,26)_ = 9.63	*p* = 0.005
	7*d*	Males: thin spines	Two-way LMM ANOVA	Treatment: *F*_(2,13)_ = 30.63	*p* < 0.0001
	7*d*	Females: thin spines	Two-way LMM ANOVA	Intake × treatment: *F*_(2,13)_ = 3.92	*p* = 0.047
	7*d*	Sal SA: high-Alc vs water	Tukey’s *post hoc*	*t*_(13)_ = 3.67	*p* = 0.008
	7*d*	Ket SA: low-Alc vs water	Tukey’s *post hoc*	*t*_(15)_ = 5.25	*p* = 0.0004
	7*d*	Ket SA: high-Alc vs water	Tukey’s *post hoc*	*t*_(15)_ = 4.72	*p* = 0.001
	7*d*	Water: Ket SA vs Sal SA	Tukey’s *post hoc*	*t*_(15)_ = −3.47	*p* = 0.004
q	7*e*	Mushroom spines	Three-way LMM ANOVA	Sex × intake × treatment: *F*_(2,26)_ = 8.82	*p* = 0.001
	7*e*	Males vs females: high-Alc, Ket SA	Tukey’s *post hoc*	*t*_(26)_ = −6.22	*p* < 0.0001
	7*e*	Males vs Females: low-Alc, Ket SA	Tukey’s *post hoc*	*t*_(26)_ = 2.6	*p* = 0.01
	7*e*	Males: mushroom spines	Two-way LMM ANOVA	Intake × treatment: *F*_(2,13)_ = 12.08	*p* = 0.001
	7*e*	Water: Ket SA vs Sal SA	Tukey’s *post hoc*	*t*_(13)_ = 5.21	*p* = 0.0005
	7*e*	Low-Alc: Ket SA vs Sal SA	Tukey’s *post hoc*	*t*_(13)_ = 7.16	*p* < 0.0001
	7*e*	High-Alc: Ket SA vs Sal SA	Tukey’s *post hoc*	*t*_(13)_ = 0.21	*p* = 0.83
	7*e*	Females: mushroom spines	Two-way LMM ANOVA	Treatment: *F*_(1,13)_ = 72.6	*p* < 0.0001
r	7*f*	Stubby spines	Three-way LMM ANOVA	Sex × intake × treatment: *F*_(2,26)_ = 0.02	*p* = 0.97
s	8*a*	Total × alcohol (%pref)	Linear regression	Males: *R* ^2^ = 0.54 Females: *R* ^2^ = 0.49	*p* = 0.007 *p* = 0.01
	8*b*	Thin × alcohol (%pref)	Linear regression	Males: *R* ^2^ = 0.35 Females: *R* ^2^ = 0.35	*p* = 0.04 *p* = 0.04
	8*c*	Mushroom × alcohol (%pref)	Linear regression	Males: *R* ^2^ = 0.06 Females: *R* ^2^ = 0.1	*p* = 0.44 *p* = 0.31
t	8*d*	Total × Cum. infusions	Linear regression	Males: *R* ^2^ = 0.17 Females: *R* ^2^ = 0.09	*p* = 0.08 *p* = 0.21
	8*e*	Thin × Cum. infusions	Linear regression	Males: *R* ^2^ = 0.3 Females: *R* ^2^ = 0.007	*p* = 0.01 *p* = 0.73
	8*f*	Mushroom × Cum. infusions	Linear regression	Males: *R* ^2^ = 0.18 Females: *R* ^2^ = 0.56	*p* = 0.07 *p* = 0.003

Summary of analyses performed on behavioral, morphologic, and correlational data. Each comparison is indicated by lettering in the far-left column (column 1). Figure column represents each corresponding to that figure or panel for that comparison. Comparison column represents the dependent variable being measured as well as individual comparisons examined with *post hoc* tests. Type of test indicates the analysis performed on that particular dataset. Statistic column indicates sample size, df, and *F* statistic for each comparison. Statistical interactions and/or main effects observed are indicated within this column. Confidence interval (CI) set at 95% lists the corresponding *p* values for each statistic, and any comparison *p* < 0.05 was considered statistically significant. NA, Not applicable; Cum., cumulative; LMM ANOVA, linear mixed-models ANOVA. %pref, percentage of preference; F, female; M, male.

## Results

### High versus low alcohol intake

Before the start of ketamine self-administration, male and female rats consumed alcohol for 3 weeks (Fig. 1*a*, time line). Three-way linear mixed-model ANOVA performed on alcohol intake (in grams per kilogram) during the first 3 weeks revealed a significant main effect of sex, indicating that female rats consume a higher dose of alcohol than males (main effect of sex: *F*_(1,59)_ = 48.42, *p* < 0.0001; mean ± SEM: males, 4.89 ± 0.2 g/kg; females, 8.81 ± 0.33). Additionally, three-way linear mixed-model ANOVAs performed on alcohol preference (percentage) during the first 3 weeks revealed a significant main effect of sex, suggesting that female rats display a higher preference for alcohol than males as well (main effect of sex: *F*_(1,58)_ = 17.61, *p* < 0.0001; mean ± SEM: males, 19.69 ± 0.77 g/kg; females, 28.69 ± 1.14). To investigate how varying levels of alcohol consumption between rats might differentially affect ketamine self-administration, rats were divided into high- or low-alcohol drinkers. Average intake and preference during week 3 was used to determine the high versus low subgroups before beginning ketamine self-administration on week 4 (Fig. 1*b*,*c*). Unpaired *t* tests performed on week 3 average alcohol intake and preference revealed that female rats show elevated levels of alcohol consumption, and, as such, we divided rats into high- versus low-alcohol subgroups separately within each sex (males vs females: intake: *t*_(60)_ = 4.66, *p* < 0.0001; preference: *t*_(60)_ = 2.74, *p* = 0.008; Fig. 1*b*,*c*). A median split on average alcohol intake (in grams per kilogram) and preference (percentage) during the third week of drinking for males resulted in a cutoff of 4.3 g/kg and 16% preference, whereas for females the cutoff was 6.9 g/kg and 23% preference (Fig. 1*b*,*c*). Rats consuming alcohol below these criteria were placed in the low-alcohol subgroup, and rats above the criteria were placed in the high-alcohol subgroup.

### Body weights

Body weights were measured before each drinking session throughout the experiment. Before the start of the IA2BC20% paradigm, a significant main effect of sex was observed in weight; however there was no intake × treatment interaction within each sex, suggesting that body weights were similar across groups within each sex (three-way mixed-model ANOVA: main effect of sex: *F*_(1,93)_ = 736.83, *p* < 2e^−16^; mean ± SEM: males, 363 ± 4.29; females, 232 ± 1.94). Body weights measured during the 3 week period before self-administration yielded no significant group differences, though main effects of sex and sessions were observed, indicating that males weigh significantly more than females but that intake does not impact weight gain over time (four-way mixed-model ANOVA: main effect of sex: *F*_(1,93)_ = 946.9, *p* < 0.0001; main effect of sessions: *F*_(8,744)_ = 901.53, *p* < 0.0001; mean ± SEM: males, 410 ± 2.04; females, 250 ± 0.84). Following catheter surgery, all rats were exposed to one drinking session before the start of self-administration, and unpaired *t* tests revealed that body weights were similar between saline and ketamine subgroups [males: water: *t*_(20)_ = 1.48, *p* = 0.15 (Sal: 480 ± 10.42 vs Ket: 455 ± 13.51); low-Alc: *t*_(14)_ = 0.96, *p* = 0.35 (Sal: 465 ± 13.89 vs Ket: 488 ± 19.39); High-Alc: *t*_(13)_ = 0.75, *p* = 0.46 (Sal: 464 ± 9.29 vs Ket: 478 ± 15.8); females: water: *t*_(20)_ = 0.76, *p* = 0.46 (Sal: 279 ± 5.93 vs Ket: 272 ± 7.2); low-Alc: *t*_(14)_ = 0.27, *p* = 0.79 (Sal: 273 ± 6.7 vs Ket: 270 ± 8.27); high-Alc: *t*_(12)_ = 1.3, *p* = 0.23 (Sal: 268 ± 5.42 vs Ket: 277 ± 4.63)].

A significant four-way interaction was observed when examining body weight gain throughout the experiment, and a main effect of sex indicated that female rats weigh significantly less than males throughout the experiment (sex × intake × treatment × sessions: *F*_(60, 2790)_ = 1.57, *p* = 0.004; main effect of sex: *F*_(1,93)_ = 1022.21, *p* < 0.0001). Among male rats, a significant intake × treatment × sessions interaction was observed (intake × treatment × sessions: *F*_(60,1410)_ = 1.75, *p* = 0.0004). *Post hoc* analysis revealed that while ketamine had no effect on weight gain in high- or low-alcohol intake males, it significantly reduced weight gain in water-intake male rats compared with saline self-administering controls [males: sessions 17–31 (weeks 6–10); water: *t*_(47)_ ≤ 2, *p* < 0.05 (Sal: 563 ± 12.01 vs Ket: 519 ± 15.72); low-Alc: *t*_(47)_ > 0.147, *p* < 0.05 (Sal: 540 ± 21.4 vs Ket: 556 ± 22.09); high-Alc: *t*_(47)_ > 0.177, *p* < 0.05 (Sal: 532 ± 11.35 vs Ket: 545 ± 19.15)]. In females, there was no significant intake × treatment × sessions interaction throughout the 10 week period, suggesting that weight gain was similar across all groups [three-way linear mixed-model ANOVA: *F*_(60,1380)_ = 0.54, *p* = 0.99; females: sessions 17–31 (weeks 6–10); water-Sal: 306 ± 6.8 vs Ket: 295 ± 6.5; low-Alc_Sal: 299 ± 6.7 vs Ket: 292 ± 8.2; high-Alc_Sal: 297 ± 7.9 vs Ket: 301 ± 4.6]. Together, these data indicate that ketamine reduces body weight gain in male rats, but not in female rats, and that alcohol, regardless of intake amount, rescues these effects.

### The effect of alcohol on ketamine self-administration during acquisition

**Figure 2.**
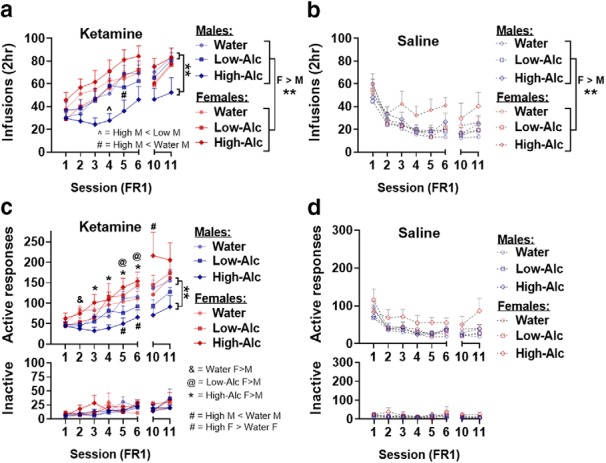
Ketamine acquisition under the FR1 schedule of reinforcement is reduced in high-alcohol intake male rats, but not female rats. ***a***, ***b***, Number of ketamine or saline infusions under an FR1 schedule of reinforcement during 2 h sessions in male and female rats, respectively. ***a***, Infusions were significantly decreased in high-alcohol intake male rats (*n* = 8) in session 4 compared with low-alcohol intake rats (*n* = 8) and session 5 to water-intake rats (*n* = 11). Female rats self-administered more ketamine than males, and high-alcohol intake female rats were significantly higher than high-alcohol intake male rats for the final two FR1 sessions (SA sessions 10–11). ***b***, No intake differences were observed in saline self-administering rats of either sex. ***c***, ***d***, Number of active and inactive responses during FR1 sessions in male and female rats. Responses include the number of nose pokes rewarded and unrewarded during the 20 s timeout period. ***c***, High-alcohol intake male rats decreased active responses in session 5 compared with water-intake males and session 6 compared with low-alcohol intake males, and an overall reduction was observed during the final two FR1 sessions (SA sessions 10–11). Water-intake females (*n* = 11) showed a significant increase in active responses compared with males in session 2, while high-alcohol intake females (*n* = 7) displayed this sex difference from sessions 3 to 6 and low-alcohol (*n* = 8) from sessions 5 to 6. ***d***, Intake did not affect responding in the saline groups. The *, #, @, &, ^ *p* < 0.05 symbols represent either within- or between-sex differences (indicated in ***a*** and ***c***). Data are represented as the mean ± SEM infusions or responses for ketamine (0.5 mg/kg/infusion) or saline. Saline self-administration male and female rats with water intake (*n* = 11), low alcohol intake (*n* = 8), and high alcohol intake (*n* = 7). Low-alcohol intake female rats that self-administered ketamine (*n* = 8). **p < 0.01.

Given previous reports demonstrating that ketamine treatment reduced alcohol intake and withdrawal-related behaviors (Sabino et al., 2013a; Holleran et al., 2016; Rezvani et al., 2017), we hypothesized that alcohol would similarly alter ketamine addictive-like behaviors. There were no differences observed in sucrose pellet training data, suggesting that all groups similarly learned the task (four-way linear mixed-model ANOVA: *F*_(8,364)_ = 0.85, *p* = 0.56; males: Sal: 49 ± 2.65 vs Ket: 47 ± 2.44; females: Sal: 53 ± 2.36 vs Ket: 55 ± 2.19). During FR1 acquisition sessions, female rats showed significantly higher rates of infusions compared with male rats (main effect of sex: *F*_(1,93)_ = 9.02, *p* = 0.0034; Fig. 2*a*,*c*). In male rats, there was a significant intake × treatment × sessions interaction between infusions during the first six FR1 sessions (*F*_(10,230)_ = 2.3, *p* = 0.014; Fig. 2*a*). *Post hoc* analyses revealed significant reductions in ketamine infusions among high-alcohol intake male rats compared with low-alcohol intake males on SA session 4, and water-intake males on session 5 (high-Alc vs low-Alc, session 4: *t*_(46)_ = 2.8, *p* = 0.02; high-Alc vs water, SA sessions 5: *t*_(46)_ = −3.13, *p* = 0.008; Fig. 2*a*). Furthermore, statistical trends toward a significant reduction in ketamine infusions among high-alcohol intake male rats compared with water intake males were observed in sessions 3, 4, and 6 (high-Alc vs water, SA sessions 3, 4, and 6: *t*_(46)_ = −2.17, −2.25, −2.19; *p* = 0.07, 0.07, 0.08, respectively; Fig. 2*a*). No effects of alcohol intake were observed among saline self-administration male rats. In female rats, ketamine was self-administered at significantly increased rates of infusions compared with saline, although intake had no effect on ketamine or saline self-administration (treatment × sessions: *F*_(5,235)_ = 38.03, *p* < 0.0001). Together, high-alcohol intake male rats displayed an attenuation of ketamine self-administration; however, ketamine self-administration was not affected by intake in female rats.

A significant four-way interaction was observed when examining active responses during the first six FR1 sessions (sex × intake × treatment × sessions: *F*_(10,459)_ = 2.13, *p* = 0.021; Fig. 2*c*). *Post hoc* analysis revealed sex differences in the number of active responses were higher in female rats compared with males and only in ketamine groups. Water-intake females displayed significantly increased active responses at session 2 (SA session 2: *t*_(93)_ = 2.33, *p* = 0.022; Fig. 2*c*). From session 5 to 6, low-alcohol intake female rats displayed significantly increased numbers of active responses compared with males (SA sessions 5–6: *t*_(93)_ = 2.18, 2.62; *p* = 0.032, 0.01, respectively; Fig. 2*c*). From sessions 3–6, active responses were significantly increased among high-alcohol intake female rats compared with high-alcohol intake male rats (SA sessions 3–6: *t*_(93)_ = 3.51, 3.53, 4.54, 4.49; *p* = 0.0007, 0.0007, 0.0001, 0.0001, respectively; Fig. 2*c*). Together, these data suggests that, overall, female rats display higher active responding compared with males, and that this effect persists throughout FR1 acquisition sessions in the alcohol groups, but not in the water groups.

Active responses during acquisition showed significant reductions in responding in the high-alcohol male group compared with the water-intake group on sessions 5–6 (intake × treatment × sessions: *F*_(10,228)_ = 3.13, *p* = 0.0009; high-Alc vs water, sessions 5 and 6: *t*_(46)_ = −3.59, −2.95, *p* = 0.002, 0.01; Fig. 2*c*). Furthermore, active responses during acquisition were trending toward a significant reduction in high-alcohol intake male rats compared with low-alcohol intake males in session 4 (high-Alc vs low-Alc, SA session 4: *t*_(46)_ = −2.37, *p* = 0.057; Fig. 2*b*).

Within female rats, active responses during the first six sessions were higher among ketamine than saline groups, and intake did not impact number of responses for ketamine or saline (treatment × sessions: *F*_(5,231)_ = 26.44, *p* < 0.0001; Fig. 2*c*,*d*). While inactive responses were significantly higher among females than males overall, and no intake or treatment differences were observed in either sex (main effect of sex: *F*_(1,94)_ = 4.1, 0.04; Fig. 2*c*,*d*).

Together, these data show the existence of sex differences in ketamine self-administration and demonstrate that chronic alcohol intake does not alter ketamine self-administration in female rats but reduces it in male rats.

### The effect of alcohol on motivation to self-administer ketamine

**Figure 3.**
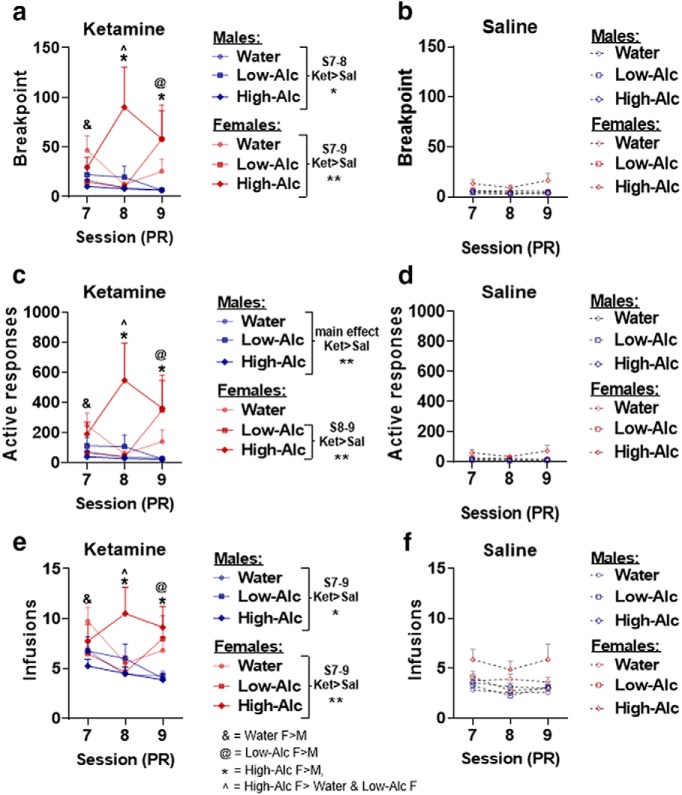
Motivation to self-administer ketamine is not maintained in either sex, but high-alcohol intake female rats show enhanced motivation during the second session. ***a***, ***b***, PR break-point data in rats that self-administered ketamine and saline. In the ketamine groups, water-intake females (*n* = 11) have significantly higher break points than males on the first session (SA session 7). High-alcohol intake females (*n* = 7) have significantly increased break points in SA sessions 8–9, while low-alcohol intake females (*n* = 8) have increased break points in SA session 9. The male groups that self-administered ketamine have increased break points compared with saline only for the first two sessions (SA sessions 8–9) before they decrease, whereas water-intake females are only significantly higher than saline in SA session 7, low-alcohol only in SA session 9, and high-alcohol in SA sessions 8 and 9. ***c***, ***d***, Number of active responses during the PR sessions for rats that self-administered ketamine or saline. Data from active responses parallel break-point data with the exception that water-intake females that self-administered ketamine did not show differences from the saline groups. ***e***, ***f***, Number of ketamine or saline infusions under a PR schedule of reinforcement. Sessions ended after failure to achieve the next ratio in a 1 h time period. Males and females self-administered significantly more ketamine infusions compared with saline across all three sessions. High-alcohol intake females took significantly higher rates of ketamine compared with low-alcohol or water-intake females. *, #, &, @, ^ *p* < 0.05 symbols represent either within- or between-sex differences (indicated in ***e***). Data are represented as the mean ± SEM break point and ketamine (0.5 mg/kg/infusion) or saline infusions. Saline self-administration male and female rats with water intake (*n* = 11), low alcohol intake (*n* = 8), and high alcohol intake (*n* = 7). **p < 0.01.

To assess the effect that alcohol intake has on motivation to self-administer ketamine, rats were subjected to three PR trials following the acquisition of ketamine or saline self-administration (Fig. 1*a*, time line). Four-way mixed-model ANOVA revealed a significant four-way interaction among sex, intake, treatment, and sessions when examining PR break point (*F*_(4,186)_ = 3.6, *p* = 0.0074; Fig. 3*a*,*b*). During the first PR session, water-intake female rats displayed significantly increased break points for ketamine but not saline compared with males (SA session 7: *t*_(93)_ = 2.21, *p* = 0.029; Fig. 3*a*). For the low-alcohol groups that self-administered ketamine, female rats displayed significantly increased break points on the final PR session (SA session 9: *t*_(93)_ = 3.29, *p* = 0.0014; Fig. 3*a*). High-alcohol intake female rats that self-administered ketamine displayed increased break points compared with males in the same group for the final two PR sessions (SA sessions 8–9: *t*_(93)_ = 5.27, 3.32; *p* = 0.0001, 0.0013, respectively; Fig. 3*a*). Within male rats, a treatment × sessions interaction revealed that the ketamine groups displayed significantly increased break points compared with the saline groups during the first two PR sessions, but not the last one (treatment × sessions: *F*_(2,92)_ = 3.21, *p* = 0.045; SA sessions 7–8: *t*_(46)_ = 4.08, 2.94; *p* = 0.0002, 0.0052, respectively; Fig. 3*a*,*b*). Within female rats, a significant intake × treatment × sessions interaction was observed (*F*_(4,94)_ = 3.67, *p* = 0.008; Fig. 3*a*). *Post hoc* analysis revealed that water-intake females that self-administered ketamine showed significantly higher break points compared with saline on the first PR session but not the second or third (SA session 7: *t*_(47)_ = 2.25, *p* = 0.029; Fig. 3*a*,*b*). Low-alcohol intake female rats displayed significantly increased break points compared with saline only in the third PR session (SA session 9: *t*_(47)_ = 2.52, *p* = 0.015; Fig. 3*a*,*b*). High-alcohol intake female rats in the ketamine group were significantly higher than saline in the second PR session only, and a statistical trend toward significance was noted in the third session (SA session 8–9: *t*_(47)_ = 3.67, 1.89; *p* = 0.0006, 0.06, respectively; Fig. 3*a*,*b*). Furthermore, on the second session, the high-alcohol intake female rats that self-administered ketamine displayed significantly increased break points compared with the water- and low-alcohol intake females (SA session 8: high-Alc vs water: *t*_(47)_ = 3.94, *p* = 0.0008; high-Alc vs low-Alc: *t*_(47)_ = 3.78, *p* = 0.012; Fig. 3*a*).

Similar to break-point data, there was a significant four-way interaction when examining the number of active responses during the PR sessions (sex × intake × treatment × session: *F*_(4,185)_ = 3.25, *p* = 0.013; Fig. 3*c*,*d*). *Post hoc* analyses revealed that female rats displayed significantly increased active responses compared with males during the first session for the water group, the third session for the low-alcohol group, and the final two PR sessions for the high-alcohol groups (SA session 7, water: *t*_(93)_ = 1.99, *p* = 0.049; SA session 9, low-Alc: *t*_(93)_ = 3.27, *p* = 0.0015; SA sessions 8–9, high-Alc: *t*_(93)_ = 5.2, 3.43, *p* = 0.0001, 0.0009; Fig. 3*c*). Within males, the ketamine groups were significantly higher than the saline groups (main effect of treatment: *F*_(1,46)_ = 10.43, *p* = 0.0023; Fig. 3*c*,*d*). Within females, the water-intake group self-administering ketamine did not show any significant differences in active responding compared with saline intake, though there was a trend toward statistical significance on the first PR session (intake × treatment × sessions: *F*_(4,94)_ = 3.25, *p* = 0.015; SA session 7: *t*_(47)_ = 1.92, *p* = 0.054; Fig. 3*c*,*d*). Females self-administering ketamine in the low-alcohol group showed significantly higher active responses compared with saline on the third PR session, while females in the high-alcohol group showed significantly higher active responses on the second and third PR session (SA session 9, low-Alc: *t*_(47)_ = 2.48, *p* = 0.017; SA sessions 8–9, high-Alc: *t*_(47)_ = 3.65, 2.05, *p* = 0.0007, 0.047, respectively; Fig. 3*c*,*d*). Additionally, high-alcohol intake females within the ketamine group showed significantly higher active responses compared with the water- and low-alcohol intake groups (SA session 8: water: *t*_(47)_ = 3.88, *p* = 0.0009; low-Alc: *t*_(47)_ = 3.76, *p* = 0.0014; Fig. 3*c*,*d*).

When examining the number of infusions within the PR trials, a sex × intake × treatment × sessions interaction was identified (*F*_(4,186)_ = 2.95, *p* = 0.022; Fig. 3*e*,*f*). Among water-intake rats self-administering ketamine, but not saline, females showed significantly higher rates of infusions compared with males on the first PR session (SA session 7: *t*_(47)_ = 2.26, *p* = 0.026; Fig. 3*e*). Among low-alcohol intake rats, females self-administered significantly more ketamine infusions than males on the final session (PR session 9: *t*_(47)_ = 2.69, *p* = 0.0084; Fig. 3*e*), and high-alcohol intake female rats also self-administered significantly higher ketamine infusions than males (PR session 8–9: *t*_(47)_ = 4.04, 3.54, *p* = 0.0001, 0.0006; Fig. 3*e*). In male rats, the ketamine group displayed significantly higher infusions compared with saline across each session (treatment × sessions: *F*_(2,92)_ = 4.32, *p* = 0.016; session 7–9: *t*_(46)_ = 5.48, 4.17, 2.09, *p* = 0.0001, 0.0001, 0.04, respectively; Fig. 3*e*,*f*). In female rats, the water-intake group self-administered significantly more ketamine than saline on the first and third PR sessions (intake × treatment × sessions: *F*_(4,94)_ = 4.48, *p* = 0.002; SA sessions 7 and 9: *t*_(47)_ = 3.82, 2.03; *p* = 0.0004, 0.049, respectively; Fig. 3*e*,*f*). Female rats in the high- and low-alcohol intake groups self-administered significantly more ketamine than saline on the second and third PR sessions, respectively (SA session 8, high-Alc: *t*_(47)_ = 2.96, *p* = 0.005; SA session 9, low-Alc: *t*_(47)_ = 2.71, *p* = 0.009; Fig. 3*e*,*f*). Furthermore, high-alcohol intake female rats showed significantly increased ketamine infusions compared with low-alcohol and water-intake rats (SA session 8: high-Alc vs water: *t*_(47)_ = 2.9, *p* = 0.02; high-Alc vs low-Alc: *t*_(47)_ = 3.19, *p* = 0.007; Fig. 3*e*). Together, these data suggest that female rats exhibit greater motivation to self-administer ketamine compared with male rats. Also, the high-alcohol intake female group exhibited increased motivation to self-administer ketamine compared with the water- and low-alcohol intake groups of female rats.

### The effects of alcohol on post-PR training

Rats were subjected to two final FR1 sessions following PR sessions to stabilize ketamine self-administration. During these sessions, there was no significant sex difference in infusions, but a sex × intake interaction revealed that high-alcohol intake females self-administered significantly more ketamine than high-alcohol intake males (sex × intake: *F*_(2,93)_ = 3.45, *p* = 0.036; high-Alc: *t*_(93)_ = 3.05, *p* = 0.003; Fig. 2*a*). Interestingly, this sex difference was not observed for the water- and low-alcohol intake groups self-administering ketamine during the last FR1 sessions (water: *t*_(93)_ = 0.365, *p* = 0.72; low-Alc: *t*_(93)_ = −0.301, *p* = 0.76; Fig. 2*a*). To confirm that infusions had stabilized during the final two FR1 sessions, a four-way mixed-model ANOVA was used to analyze infusions during the final two sessions of FR1 acquisition (SA sessions 5–6) and the two FR1 sessions after PR training (SA sessions 10–11). In rats of both sexes, there was no significant interaction, suggesting stable rates of infusions during the final two sessions (intake × treatment × sessions: males: *F*_(6,138)_ = 0.69, *p* = 0.66; females: *F*_(6,141)_ = 0.56, *p* = 0.76; Fig. 2*a*,*b*). In summary, rats of both sexes display similar rates of infusion during the final two FR1 sessions compared with last 2 d of acquisition before PR sessions.

During the final two FR1 sessions, female rats showed increased levels of active responding compared with males (four-way mixed-model ANOVA, main effect of sex: *F*_(1,93)_ = 9.93, *p* = 0.002; Fig. 2*c*,*d*). Active responding within male rats showed that responding for ketamine was reduced in the high-alcohol group compared with water (intake × treatment: *F*_(2,46)_ = 3.65, *p* = 0.034; Ket: high-Alc vs water: *t*_(46)_ = −3.17, *p* = 0.008, Fig. 2c). High-alcohol intake female rats displayed significantly increased responding for ketamine compared with the water-intake group during SA session 10, though this effect was abolished during the final FR1 session, suggesting that the increase may be an artifact of post-PR training (intake × treatment × sessions: *F*_(2,46)_ = 4.12, *p* = 0.02; SA session 10, Ket: high-Alc vs water: *t*_(46)_ = 2.77, *p* = 0.022; Fig. 2*c*). Three-way mixed-model ANOVAs within each sex were used to compare the final two sessions of acquisition (SA sessions 5–6) with the final two FR1 sessions (SA sessions 10–11) to confirm stable levels of responding during the final FR1 sessions, and there was no intake × treatment × sessions interaction observed in either sex, suggesting stable levels of active responding (males: *F*_(6,138)_ = 0.15, *p* = 0.98; females: *F*_(6,139)_ = 1.26, *p* = 0.28; Fig. 2*c*,*d*). Furthermore, inactive responding during the final two FR1 sessions was similar across all groups within both sexes (intake × treatment × sessions: males: *F*_(2,45)_ = 2.7, *p* = 0.07; females: *F*_(2,45)_ = 0.99, *p* = 0.38; Fig. 2*c*,*d*). Together, both sexes show stable levels of responding after PR training. While high-alcohol intake female rats show increased responding during SA session 10, the effect disappears by SA session 11 and as such, we believe that this heightened level of responding is a result of the transition from PR training.

### The effect of alcohol on incubation of ketamine craving

**Figure 4.**
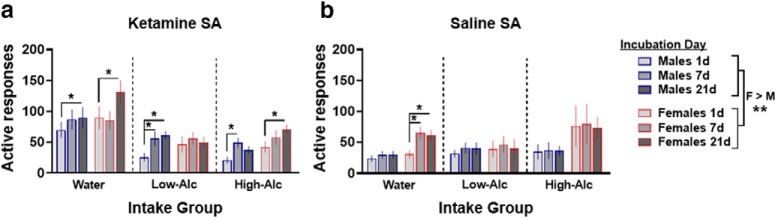
Incubation of ketamine craving develops in all groups except low-alcohol intake female rats. ***a***, ***b***, Active responses during 2 h FR1 sessions 1, 7, and 21 d into the ketamine abstinence period in male and female rats. Drug-paired cues were identical, but active responses yielded no drug infusion. Female rats displayed increased levels of active responding compared with males, regardless of ketamine or saline self-administration. ***a***, In male rats, active responses within the ketamine groups (water, *n* = 11; low-Alc, *n* = 8; high-Alc, *n* = 8) increased over the 21 d period. In female rats, those that self-administered ketamine (water, *n* = 11; low-Alc, *n* = 7; high-Alc, *n* = 7) that were considered water- and high-alcohol intake rats increased responding over time; however, low-alcohol intake female rats did not. ***b***, In male rats, intake did not affect active responding in the saline groups (water, *n* = 11; low-Alc, *n* = 8; high-Alc, *n* = 7). Water-intake female rats that self-administered saline increased responding over time while the alcohol groups did not (water, *n* = 9; low-Alc, *n* = 8; high-Alc, *n* = 7). *p < 0.05, **p < 0.01. Data are expressed as the mean ± SEM active responses.

To assess alcohol effects on the incubation of ketamine craving, rats underwent a 21 d period of abstinence from ketamine or saline, but were maintained on alcohol and tested for cue-induced ketamine seeking 1, 7, and 21 d after the final self-administration session. An overall main effect of sex was observed when examining active responses, indicating that female rats displayed significantly higher rates of active responses (*F*_(1,85)_ = 9.81, *p* = 0.0024; Fig. 4*a*,*b*). In male rats, active responding was significantly higher in ketamine self-administering rats compared with saline on withdrawal days 7 and 21, but not on withdrawal day 1 (treatment × sessions: *F*_(2,88)_ = 3.79, *p* = 0.026; withdrawal day 7 and 21: *t*_(48)_ = 3.44, 3.43, *p* = 0.0012, 0.0013, respectively; Fig. 4*a*,*b*). However, active responding among the ketamine group was significantly higher than saline in the water group (intake × treatment: *F*_(2,44)_ = 5.44, *p* = 0.008; Ket vs Sal: water: *t*_(44)_ = 4.76, *p* < 0.0001; low-Alc: *t*_(44)_ = 0.76, *p* = 0.45; high-Alc: *t*_(44)_ = 0.001, *p* = 0.99; Fig. 4*a*,*b*). Furthermore, water-intake male rats that self-administered ketamine displayed significantly higher rates of active responding compared with low- and high-alcohol intake rats, suggesting that alcohol intake may be associated with reduced ketamine craving (intake × treatment: *F*_(2,44)_ = 5.44, *p* = 0.008; high-Alc vs water: *t*_(44)_ = −3.58, *p* = 0.002; low-Alc vs water: *t*_(44)_ = −2.77, *p* = 0.022; Fig. 4*a*). *Post hoc* analyses revealed that in male rats that drank water only, active responding for cues significantly increased from day 1 to day 21 in the ketamine self-administration group but not in the saline group (water_Ket, day 1 vs 21: *t*_(91)_ = 2.52, *p* = 0.03; Fig. 4*a*,*b*). Furthermore, a trend toward statistical significance was identified in water-intake males that self-administered ketamine from day 1 to day 7 (*t*_(91)_ = 2.12, *p* = 0.09; Fig. 4*a*). Low-alcohol intake male rats that self-administered ketamine, but not saline, displayed a significant increase in active responding on day 7 versus day 1 and day 21 versus day 1 (low-Alc_Ket: day 1 vs 7: *t*_(91)_ = 3.44, *p* = 0.0025; day 1 vs 21: *t*_(91)_ = 4.05, *p* = 0.0003; Fig. 4*a*). In high-alcohol intake male rats that self-administered saline, active responses did not change over the 21 d period, while those that self-administered ketamine displayed significantly increased responding on day 7 compared with day 1 (high-Alc_Ket: day 1 vs 7: *t*_(91)_ = 2.72, *p* = 0.02; Fig. 4*a*). Furthermore, a trend toward a statistical increase in active responding in high-alcohol intake males that previously self-administered ketamine was observed on day 1 vs 21 (high-Alc_Ket: day 1 vs 21: *t*_(91)_ = 2.16, *p* = 0.07; Fig. 4*a*).

Among ketamine female rats, active responding was significantly increased in water-intake rats compared with low- and high-alcohol intake rats, suggesting that alcohol is associated with a reduction in ketamine craving (intake × treatment: *F*_(2,41)_ = 3.93, *p* = 0.027; high-Alc vs Water: *t* = −2.55, *p* = 0.038; low-Alc vs water: *t* = −2.9, *p* = 0.016; Fig. 4*a*). Furthermore, active responding was significantly increased for ketamine compared with saline craving in the water group but not in either alcohol intake group of female rats (intake × treatment: *F*_(2,41)_ = 3.93, *p* = 0.028; Ket vs Sal: water: *t*_(41)_ = 2.93, *p* = 0.006; low-Alc: *t*_(41)_ = 0.36, *p* = 0.72; high-Alc: *t*_(41)_ = −1.14, *p* = 0.26; Fig. 4*a*,*b*). Female rats that self-administered ketamine displayed an increase in active responding over time, whereas saline-intake females did not (main effect of sessions: Ket: *F*_(2,44)_ = 3.35, *p* = 0.04; Sal: *F*_(2,38)_ = 1.97, *p* = 0.15; Fig. 4*a*,*b*). *Post hoc* analyses revealed that water-intake female rats that self-administered saline displayed increases in active responding from day 1 to 7 and from day 1 to 21, whereas female rats that self-administered ketamine displayed increases in active responding from day 1 to 21 (water_Sal: day 1 vs 7: *t*_(82)_ = 2.8, *p* = 0.01; day 1 vs 21: *t*_(82)_ = 2.8, *p* = 0.01; water_Ket: day 1 vs 21: *t*_(82)_ = 2.8, *p* = 0.01; Fig. 4*a*,*b*). Low-alcohol intake female rats did not show increases in active responding throughout the 21 d period, regardless of drug group. High-alcohol intake female rats that self-administered ketamine, but not saline, displayed increased active responding from day 1 to 21 (high-Alc_Ket: *t*_(82)_ = 2.52, *p* = 0.04; Fig. 4*a*,*b*). Similar to males, alcohol intake may be contributing to reduced cue-induced reinstatement in high- and low-intake female rats. Together, these results suggest that ketamine self-administration produces incubation of ketamine craving in both sexes except in the low-alcohol intake female group.

### The effect of ketamine self-administration on alcohol intake and preference

**Figure 5.**
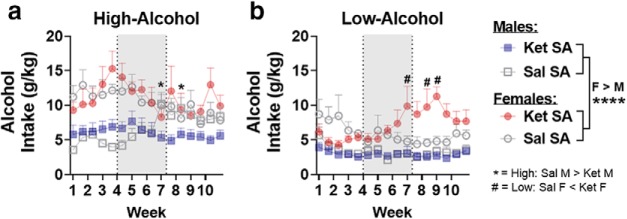
Ketamine decreases alcohol intake in high-alcohol intake male rats while increasing it in low-alcohol intake females. ***a***, ***b***, Alcohol intake (g/kg/24 h) over a 10 week period, with self-administration of ketamine or saline occurring from week 4 to 7 (indicated by shaded rectangles). ***a***, In high-alcohol intake male rats, self-administration of ketamine (*n* = 8) blocks the escalation in alcohol intake observed in saline rats (*n* = 7). Ketamine had no effect on high-alcohol intake female rats (Sal, *n* = 7; Ket, *n* = 8). ***b***, In low-alcohol intake male rats, ketamine had no effect on alcohol intake (Sal, *n* = 8; Ket, *n* = 8). In low-alcohol intake female rats, ketamine self-administration enhanced alcohol intake compared with saline from session 19 (week 7) to 28 (week 9; Sal, *n* = 8; Ket, *n* = 8). *p < 0.05. Data are expressed as the mean ± SEM alcohol preference. *, #*p* < 0.05; symbols represent either within- or between-sex differences (indicated on Fig. 5*a*,*b*), ****p < 0.0001.

To assess the effect that ketamine had on alcohol consumption, the dose of alcohol consumed (intake) as well as the preference for alcohol over water (percentage) across the 10 week period were analyzed. Female rats consumed significantly higher doses (in grams per kilogram) of alcohol compared with males (main effect of sex: *F*_(1,53)_ = 83.87, *p* < 0.0001; Fig. 5). High-alcohol intake male rats that self-administered ketamine displayed a significant reduction in alcohol intake compared with rats self-administering saline (intake × treatment × sessions: *F*_(30,785)_ = 2.53, *p* < 0.0001, sessions 19 and 22: *t*_(27)_ < −2.68, −3.08; *p* = 0.019, 0.009; Fig. 5*a*). Furthermore, there were other sessions where a statistical trend toward a significant reduction in high alcohol intake among males that self-administered ketamine was observed (sessions 18, 20, 21, 23, 25, and 29: *t*_(27)_ < −1.79; *p* < 0.1; Fig. 5*a*). Low-alcohol intake female rats displayed significant increases in alcohol intake compared with the saline group (intake × sessions: *F*_(30,760)_ = 2.5, *p* < 0.0001; treatment × sessions: *F*_(30,760)_ = 1.6, *p* = 0.022; sessions 19–20, and 23–26: *t*_(27)_ > 2.26; *p* < 0.05; sessions 21–22, and 27–28): *t*_(27)_ > 1.90; *p* < 0.1; Fig. 5*b*). Low-alcohol male rats and high-alcohol female rats did not display any ketamine-induced differences in alcohol intake (Fig. 5*a*,*b*).

**Figure 6.**
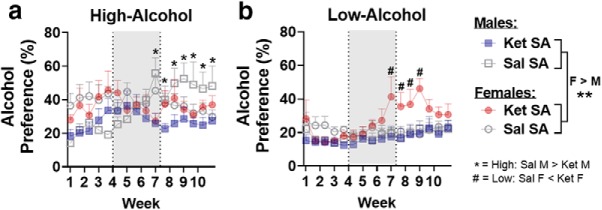
Ketamine decreases preference for alcohol in high-alcohol intake male rats while increasing it in low-alcohol intake females. ***a***, ***b***, Alcohol preference (percentage) over a 10 week period, with self-administration of ketamine or saline occurring from week 4 to 7 (indicated by shaded rectangles). ***a***, In male rats, self-administration of ketamine (*n* = 8) blocks the escalation in alcohol preference observed in high-alcohol intake saline (*n* = 7) rats. Preference was significantly attenuated from session 18 (week 6) to 31 (week 10). In high-alcohol intake female rats, ketamine had no effect on preference (Sal, *n* = 7; Ket, *n* = 8). ***b***, In low-alcohol intake male rats, ketamine had no effect on preference (Sal, *n* = 8; Ket, *n* = 8). In low-alcohol intake female rats, preference for alcohol was enhanced in rats that self-administered ketamine compared with saline from session 19 (week 7) to 28 (week 9; Sal, *n* = 8; Ket, *n* = 8). *p < 0.05. Data are expressed as the mean ± SEM alcohol preference.*, #*p* < 0.05; symbols represent either within- or between-sex differences (indicated on Fig. 5*a*,*b*), **p < 0.01.

For alcohol percentage preference, an overall main effect of sex suggested that female rats display an increased preference for alcohol compared with males (*F*_(1,53)_ = 6.19, *p* = 0.016; Fig. 6*a*,*b*). In both sexes, significant interactions among intake, treatment, and sessions were observed (males: *F*_(30,784)_ = 2.28, *p* = 0.0001; females: *F*_(30,759)_ = 1.67, *p* = 0.015). *Post hoc* analyses revealed that ketamine, but not saline self-administration, reduced alcohol preference in high-alcohol intake male rats (sessions 19–23 and 25–31): *t*_(27)_ < −2.07; *p* < 0.05; Fig. 6*a*). In low-alcohol intake female rats, ketamine increased alcohol preference compared with the saline group (sessions 19–20 and 22–26: *t*_(26)_ > 2.11; *p* < 0.05; Fig. 6*b*). Ketamine had no effect on alcohol preference in low-alcohol intake male rats or high-alcohol intake female rats (Fig. 6*a*,*b*). Altogether, these data suggest that ketamine reduces alcohol consumption specifically in high-alcohol intake male rats while increasing consumption in low-alcohol intake female rats.

### Alcohol and ketamine effects on NAc dendritic spine density

**Figure 7.**
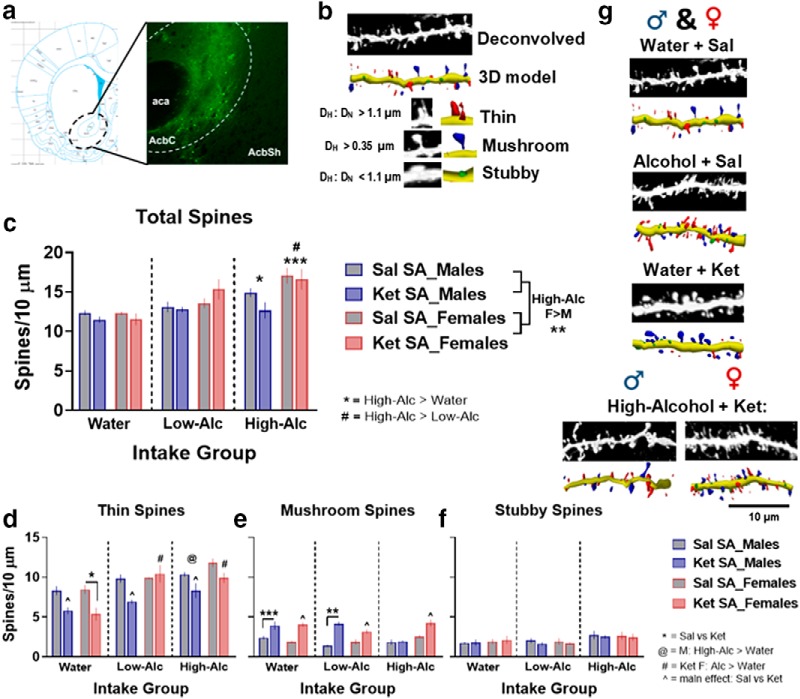
Dendritic spine density changes in the NAc are differentially impacted depending on sex and intake. ***a***, Representative image of HSV-GFP expression in the NAc. ***b***, Representative image of dendritic spines after deconvolution and 3D reconstruction featuring examples of thin, mushroom, and stubby spine shapes. D_H_, Head diameter; D_N_, neck diameter. ***c***, High alcohol intake increases total spine density in rats of both sexes compared with water-intake control rats. ***d***, High alcohol intake in male rats shows increases in thin spine density, and ketamine reduces thin spines in male rats compared with saline. Ketamine reduces thin spine density in water-intake female rats compared with saline, and both alcohol-intake groups show rescuing of these deficits. ***e***, Water intake and low alcohol intake increase mushroom spines in ketamine self-administration male rats compared with saline self-administration male rats, but this effect was not observed in high-alcohol intake males. Ketamine increased mushroom spines in female rats compared with saline, regardless of intake. ***f***, Stubby spines are unaffected by sex, intake, or treatment. ***g***, Representative images with 3D reconstructed models of dendritic spines for each treatment and intake group. *, #, @, ^ *p* < 0.05 symbols represent either within- or between-group differences (indicated in ***c*** and ***f***; *n* = 3 and 4/group; for *n* = 3, 10–12 dendrites/rat; for *n* = 4, 8–12 dendrites/rat). **p < 0.01, ***p < 0.001.

To assess whether ketamine and alcohol alter structural plasticity in the NAc, dendritic spine density was assessed 24 h after the last alcohol-/water-drinking session (3 weeks after the last FR1 ketamine/saline self-administration session and 3 d after the final incubation test). For total spines, high-alcohol intake females displayed significantly higher spine density compared with males; however, there were no sex differences in the water-intake or low-alcohol intake groups (sex × intake: *F*_(2,26)_ = 4.77, *p* = 0.017; females vs males: water: *t*_(26)_ = −0.2, *p* = 0.98; low-Alc: *t*_(26)_ = 1.99, *p* = 0.05; high-Alc: *t*_(26)_ = 4.17, *p* = 0.0003; Fig. 7c). For thin spines, females displayed significantly higher spine density compared with males (*F*_(1,26)_ = 9.63, *p* = 0.005; Fig. 7*d*). With mushroom spines, high-alcohol intake females showed significantly increased mushroom spines compared with males, whereas low-alcohol intake males showed a significant increase in mushroom spines compared with females (sex × intake × treatment: *F*_(2,26)_ = 8.82, *p* = 0.001; females vs males: high-Alc_Ket: *t*_(26)_ = 6.22, *p* < 0.0001; low-Alc_Ket: *t*_(26)_ = −2.6, *p* = 0.01; Fig. 7*e*). Interestingly, sex differences in mushroom spines were not observed among the water-intake groups that self-administered ketamine or saline or among the high- or low-alcohol intake groups that self-administered saline, suggesting that the interaction between alcohol and ketamine is affecting sex differences in mushroom spines. No sex differences were observed in stubby spine density, though a main effect of intake suggested increased stubby spines in the high-alcohol intake group (*F*_(2,33)_ = 6.12, *p* = 0.007; high-Alc vs water: *t*_(33)_ = 3.33, *p* = 0.006; high-Alc vs low-Alc: *t*_(33)_ = 3.36, *p* = 0.006; Fig. 7*f*).

In male rats, total spine density was increased in the high-alcohol intake group compared with water (main effect of intake: *F*_(2,13)_ = 3.84, *p* = 0.04; high-Alc vs water: *t*_(15)_ = 2.87, *p* = 0.02; Fig. 7*c*). Furthermore, thin spine density was increased in the high-alcohol intake group compared with the water-intake group, suggesting that thin spine increases may drive the increase in total spines (main effect of intake: *F*_(2,13)_ = 12.41, *p* = 0.008; high-Alc vs water: *t*_(15)_ = 4.13, *p* = 0.0024; Fig. 7*d*). Ketamine, however, reduced thin spines when compared with saline groups (main effect of treatment: *F*_(2,13)_ = 30.63, *p* < 0.0001; Fig. 7*d*). For mushroom spines, ketamine increased spine density in water-intake and low-alcohol intake groups compared with saline, though this was not observed in high-alcohol intake male rats (intake × treatment: *F*_(2,13)_ = 12.08, *p* = 0.001; Ket vs Sal: water: *t*_(13)_ = 4.23, *p* = 0.001; low-Alc: *t*_(13)_ = 7.16, *p* < 0.0001; high-Alc: *t*_(13)_ = 0.21, *p* = 0.83; Fig. 7*e*). Furthermore, the water-intake and low-alcohol intake groups that self-administered ketamine displayed significantly increased mushroom spines compared with the high-alcohol intake male rats (Water_Ket vs high-Alc_Ket: *t*_(13)_ = 5.21, *p* = 0.0005; low-Alc_Ket vs high-Alc_Ket: *t*_(13)_ = 5.81, *p* = 0.0002; Fig. 7*e*). Stubby spines were unchanged in male rats (intake × treatment: *F*_(2,13)_ = 0.31, *p* = 0.74; Fig. 7*f*). Together, these data indicate that in male rats, alcohol is associated with increased total and thin spines. Further, ketamine was associated with reduced thin and increased mushroom spine density, though this effect was not observed in the high-alcohol group, likely because their ketamine intake was lower than in other groups.

In female rats, total spine density was increased by alcohol since the high- and low-alcohol intake groups displayed increased spines compared with the water intake group (main effect of intake: *F*_(2,13)_ = 16.23, *p* = 0.0002, low-Alc vs water: *t*_(15)_ = 2.87, *p* = 0.02; high-Alc vs low: *t*_(15)_ = 2.64, *p* = 0.04; high-Alc vs water: *t*_(15)_ = 5.6, *p* = 0.0001, respectively; Fig. 7*c*). Thin spines were increased by high alcohol intake in the saline group compared with water intake, and among the ketamine groups, both low- and high-alcohol intake increased thin spines compared with water (intake × treatment: *F*_(2,13)_ = 3.92, *p* = 0.047; Sal: high-Alc vs water: *t*_(13)_ = 3.67, *p* = 0.008; Ket: low-Alc vs water: *t*_(15)_ = 5.25, *p* = 0.0004; high-Alc vs water: *t*_(15)_ = 4.72, *p* = 0.001; Fig. 7*d*). Furthermore, ketamine reduced thin spines in the water-intake group, though this was not observed in the high- or low-alcohol intake groups in female rats (water_Ket vs water_Sal: *t*_(15)_ = −3.47, *p* = 0.004; Fig. 7*d*). In females, mushroom spines were increased in the ketamine groups compared with saline (main effect of treatment: *F*_(1,13)_ = 72.6, *p* = 1.11e^−6^; Fig. 7*e*). No differences in stubby spines were observed among female rats (intake × treatment: *F*_(2,13)_ = 0.27, *p* = 0.77; Fig. 7*f*). Together, alcohol was associated with increases in total and thin spines in the high-alcohol group. Ketamine reduced thin spines in the water-intake group, an effect that was reversed by alcohol. Ketamine also increased mushroom type spines in all groups.

### Correlations between spines number, alcohol, and ketamine

**Figure 8.**
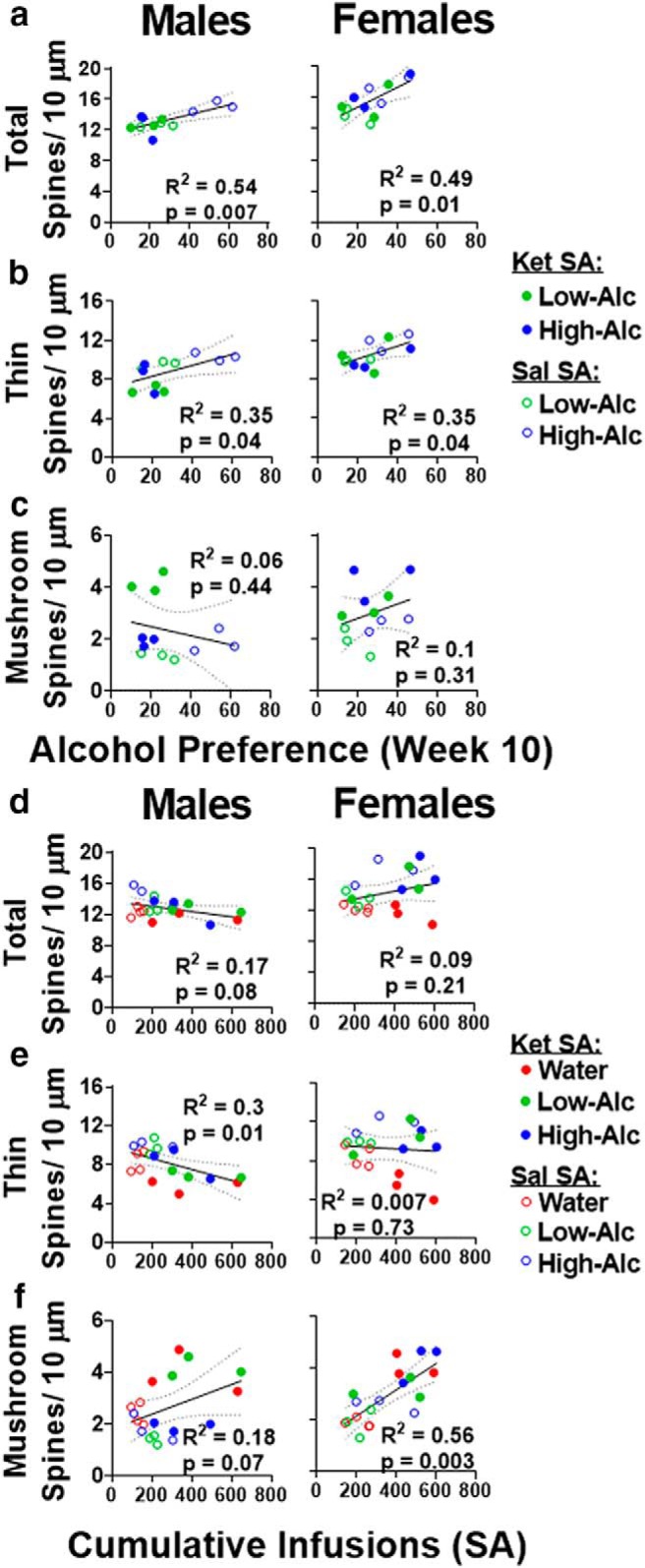
Alcohol preference is correlated with total and thin spines, while ketamine infusions are correlated with mushroom spine changes in rats of both sexes. ***a***, ***b***, Linear regression depicting positive correlations between alcohol preferences during the final week of consumption with total and thin spines, respectively. ***c***, Mushroom spines did not correlate with alcohol preference during the final week of drinking. ***d***, Total spines did not correlate with cumulative infusions during the self-administration period in either sex. ***e***, In male rats, thin spines negatively correlated with cumulative infusions during the self-administration period, but this was not observed in females. ***f***, Linear regression depicting positive correlation between mushroom spines and cumulative infusions during the self-administration sessions. *R* ^2^ and *p* values for each correlation are listed within their respective figure.

In both sexes, alcohol preference during week 10 of consumption correlated positively with total and thin spines (total, males: *R* ^2^ = 0.54, *p* = 0.007; females: *R* ^2^ = 0.49, *p* = 0.01; thin, males: *R* ^2^ = 0.35, *p* = 0.044, females: *R* ^2^ = 0.35, *p* = 0.04; Fig. 8*a*,*b*). Mushroom spines, however, did not correlate with alcohol preference during the final week of drinking (males: *R* ^2^ = 0.0.6, *p* = 0.45; females: *R* ^2^ = 0.1, *p* = 0.31; Fig. 8*c*). These data suggest that the amount of alcohol consumed is positively correlated with thin spine, but not with mushroom spines, changes in the NAc.

Total spines did not correlate with the cumulative number of infusions (FR1 and PR sessions) obtained during the self-administration period in either sex (males: *R* ^2^ = 0.17, *p* = 0.08; females: *R* ^2^ = 0.09, *p* = 0.21; Fig. 8*d*). In male rats, there was a negative correlation between thin spines and cumulative infusions, though this was not observed in female rats (males: *R* ^2^ = 0.3, *p* = 0.01; females: *R* ^2^ = 0.007, *p* = 0.73; Fig. 8*e*). Mushroom spine changes were significantly correlated with cumulative infusions in female rats and trended toward significance in male rats (males: *R* ^2^ = 0.17, *p* = 0.07; females: *R* ^2^ = 0.55, *p* = 0.0003; Fig. 8*f*). Together, these data suggest that the amount of ketamine infusions is inversely related to thin spines in male rats, but not in female rats. Furthermore, ketamine intake appears to be correlated with increased mushroom type spines in both sexes.

## Discussion

In this study, we divided rats into high- and low-alcohol intake groups and showed that ketamine self-administration is differentially regulated depending on sex and individual differences in alcohol intake. The acquisition of ketamine self-administration under an FR1 schedule of reinforcement was reduced in high-alcohol intake male rats but remained unaffected by alcohol in female rats. Motivation to self-administer ketamine under a PR schedule of reinforcement, however, was enhanced in high-alcohol intake female rats, but not in male rats. Alcohol intake was reduced in high-alcohol intake male rats that self-administered ketamine compared with saline, with a long-lasting effect that persisted at least 3 weeks after ketamine administration. Conversely, alcohol consumption in low-alcohol intake female rats was increased in rats that self-administered ketamine compared with saline for 2 weeks after ketamine self-administration. NAc total and thin spines were increased by alcohol, and alcohol preference during the final week of drinking positively correlated with these changes. After 3 weeks of forced abstinence from ketamine, there were reduced thin and increased mushroom spines in both sexes, and the cumulative number of infusions during the self-administration period positively correlated with increased mushroom spines. High-alcohol intake males, however, did not show the changes in mushroom spines that the other groups showed, possibly because they had lower ketamine intake.

In both sexes, the abuse potential of ketamine has been fairly well documented using various measures of addictive-like behaviors (De Luca and Badiani, 2011; Caffino et al., 2016, 2017, 2018; Strong et al., 2017; Wright et al., 2017, 2019; Schoepfer et al., 2019). The acquisition under FRs of reinforcement is considered to model learning to take (self-administer) a reinforcing drug and, as such, can predict risk vulnerability to drug abuse in rats (Carroll and Lac, 1993; Scofield et al., 2016). In our study, ketamine self-administration under an FR1 schedule of reinforcement was reduced in high-alcohol intake male but not female rats. This suggests that high-alcohol intake in male rats, but not in female rats, may reduce the reinforcing properties of ketamine. It should be noted, though, that infusions among rats that self-administered saline were elevated (∼20 infusions/2 h), although saline is not itself reinforcing. This is likely an artifact of the previous pellet training sessions, as these data are in line with previous studies using sucrose pellet training for operant self-administration (Wright et al., 2015). Still, studies assessing saline self-administration without the use of sucrose pellet training report ∼10–15 infusions/2 h sessions in rats, indicative of baseline responding in the absence of a reinforcing drug (Werner et al., 2018; Siemsen et al., 2019). Despite this, ketamine infusions within both sexes were significantly higher than saline infusions, demonstrating its reinforcing properties.

PRs of reinforcement assess the maximum effort that a rat is willing to put forth toward self-administering a drug and are used to model compulsive drug use and motivation (Richardson and Roberts, 1996). In this study, we show that motivation to self-administer ketamine was significantly higher than motivation to self-administer saline in both sexes, as evidenced by higher break-point, active responses, and infusions. Interestingly, in male rats, the motivation for ketamine remained stable over the three PR sessions and was only significantly higher than saline for all three sessions when examining infusions. It is possible that this effect was apparent only through infusions given that there is less variability in infusion number compared with active responses and break point, which exponentially increase with each subsequent ratio. In previous studies, however, rats subjected to PR sessions with opiates such as heroin remained stable throughout the course of the PR sessions (Roberts and Bennett, 1993). This contrasts with PR data examining psychostimulants that reliably show escalation in PR break points across many sessions (Arnold and Roberts, 1997). As discussed in the study by Scofield et al. (2016), it is likely that opiates and sedatives such as heroin and ketamine are not ideal for PR trials, given that they are known to be rewarding yet rats will not increase motivation for them over time. In female rats, we show that the motivation to self-administer ketamine was increased in the high-alcohol intake female group compared with all other groups during the second PR session. Furthermore, females self-administering ketamine show increased break point, active responses, and infusions compared with saline across all PR sessions, but further examination within each intake group revealed that the water-intake females that self-administered ketamine were higher than saline only for the first session, whereas the alcohol-intake groups that self-administered ketamine were significantly higher than saline only on the final two sessions. It is possible that the variability observed across the three sessions was the result of the estrous cycle, which is known to heavily impact PR break-point data (Roberts et al., 1989). Nonetheless, all groups of female rats similarly acquired ketamine, and the high-alcohol intake group still showed increased motivation to self-administer ketamine, suggesting that high alcohol intake may be associated with increased motivation to self-administer ketamine in female rats, but not in male rats.

We modeled relapse behaviors by using the well established incubation of craving model, where rats are re-exposed to drug-associated cues after a period of forced abstinence (Pickens et al., 2011; Venniro et al., 2016). In our study, water-intake rats of both sexes display an incubation of ketamine craving over the 21 d abstinence period. While active responses were reduced in the alcohol groups compared with water, the incubation of ketamine craving still increased over time in both alcohol-intake groups for male rats as well as high-alcohol intake female rats, suggesting that alcohol intake may contribute to reduced levels of ketamine craving. Interestingly, we did not observe incubated ketamine craving in the low-alcohol intake female rats. Given that this was the only group that increased alcohol intake during the ketamine withdrawal period, it is possible that increased alcohol consumption may reduce ketamine craving, though more work is necessary to confirm this. Furthermore, water-intake female rats that self-administered saline increased responding over the 21 d period. Though it is difficult to assess why responding for saline would increase over time, since saline lacks reinforcing properties, it is possible that this heightened responding in females was a potential incubation of sucrose craving, which was eliminated by alcohol at both low and high doses. However, this is just a speculation and will necessitate additional experiments to prove it. It is also possible that fluctuations in the estrous cycle drove the increase in responding among saline females in the water-intake group given that a previous report showed increased levels of incubated craving within estrous female rats compared with nonestrous female rats (Nicolas et al., 2019). However, we did not track the estrous cycle in the current study; therefore, it is just speculation.

Together, the current study suggests sex differences in how alcohol intake affects ketamine self-administration more significantly in male than in female rats. However, it is possible that the alcohol withdrawal period after the end of the alcohol session and before the beginning of the ketamine self-administration session contributed to the reduction in ketamine self-administration in high-alcohol intake male rats. It is also possible that a 2 h washout period before the start of self-administration was not long enough for alcohol to be cleared metabolically. This seems unlikely, though, given that most alcohol intake occurs early in the drinking period and 2 h would be sufficient to clear alcohol in adult rats (Kelly et al., 1987; Sabino et al., 2013b; Carnicella et al., 2014).

Additionally, it cannot be ruled out that the same underlying mechanisms that contribute to individual differences in alcohol intake are what led to differences in ketamine self-administration behaviors. For example, it has been shown that high versus low alcohol-preferring rats demonstrate different reactivities to the aversive properties of alcohol and that this may impact the amount of alcohol consumed (Dyr et al., 2016). Therefore, it is possible that these same underlying mechanisms are contributing to the reduced ketamine self-administration observed in high-alcohol intake male rats.

Multiple reports suggest therapeutic value for ketamine in the treatment of AUD. Indeed, an acute ketamine injection (5–20 mg/kg, i.p.) reduces alcohol intake in male and female rats (Sabino et al., 2013b; Rezvani et al., 2017; Ruda-Kucerova et al., 2018). However, ketamine-induced the reduction of alcohol intake was transient, lasting only for 2 h (Rezvani et al., 2017). This suggests that acute ketamine administration may not be effective in treating AUD, and most likely a repeated regimen of ketamine treatments may be needed in both sexes. In fact, our laboratory has previously shown that repeated exposure to ketamine has addictive-like effects across a range of doses, and that female rats are more sensitive to these effects when compared with males (Strong et al., 2017; Wright et al., 2017; Schoepfer et al., 2019). Therefore, examining the effects of administering ketamine repeatedly to treat AUD in both sexes is critical.

Preclinical studies suggest that male rats will escalate alcohol intake when given intermittent access, which is indicative of a compulsive drinking phenotype, and as such, this paradigm is used to model addictive-like behaviors associated with AUD (for review, see Carnicella et al., 2014; Ron and Barak, 2016). Much less is known regarding alcohol intake in female rats, but one study suggests different patterns of consumption between the sexes. Contrary to male rats, female rats do not escalate over time, but rather initiate alcohol consumption at higher levels than males, while maintaining stable levels of intake over time (Morales et al., 2015). The current study complements these findings in high- but not low-alcohol intake rats since low-alcohol intake rats of both sexes maintained low levels of alcohol intake throughout the 10 week period. Furthermore, our high-alcohol intake rats matched the criteria for “excessive” alcohol drinking modeled in other studies, with an intake amount above 5 g/kg, which is associated with a blood ethanol concentration of ∼80 mg/dl/30 min in rats (Sabino et al., 2013a; Carnicella et al., 2014).

In the current study, an escalation in alcohol intake and preference was observed only in high-alcohol intake male rats that were self-administering saline, an effect that was blocked in rats self-administering ketamine. In fact, ketamine-induced blockade of the escalation of alcohol intake persisted even 3 weeks after the end of ketamine self-administration. These data suggest that ketamine within this subset of male rats has long-lasting effects on reducing the addictive-like behaviors associated with AUD. It remains unknown what factors may have contributed to the ketamine-induced changes in alcohol intake, though it is possible ketamine altered tolerance to the aversive properties of alcohol, and thus, reduced motivation for consumption. Regardless, these data complement the findings from previous studies suggesting that ketamine reduces alcohol intake in male rats compared with saline (Sabino et al., 2013b; Holleran et al., 2016; Rezvani et al., 2017).

Ketamine had no effects on low-alcohol intake male rats or on high-alcohol intake female rats. Conversely, low-alcohol intake female rats displayed increased alcohol intake and preference during the ketamine self-administration period compared with the saline group, with limited effects persisting for only 2 weeks after the end of self-administration. Altogether, these data suggest that ketamine is likely a viable option for the treatment of AUD in male subjects, but not in female subjects. Further clinical studies are needed to validate our findings in men and women with AUD.

Within the mesolimbic reward pathway, the NAc is considered the “hub” of reward circuitry because it receives dopaminergic input from the ventral tegmental area and glutamatergic input from many brain regions (Mogenson et al., 1980; Heimer et al., 1997; Groenewegen et al., 1999). Therefore, to better understand how alcohol and ketamine modulate this reward pathway in both sexes, we investigated structural plasticity within the NAc. Specifically, we assessed dendritic spine density on NAc MSNs, which have been shown to dynamically change based on the lengths of drug exposure and withdrawal (Russo et al., 2010). Thin spines are characterized by a smaller spine head diameter and are considered to be more plastic given that they are new, immature spines (Sheng et al., 1994, 2008). Mushroom spines have larger head diameter due to increased AMPA receptor expression and are considered to be more excitable, stable spines (Russo et al., 2010). In general, acute withdrawal from most drugs of abuse is associated with increased thin spines, while chronic withdrawal from those drugs is associated with increased mushroom spines within the NAc (Boudreau and Wolf, 2005; Boudreau et al., 2007; Shen et al., 2009; Uys et al., 2016).

Here, we report that, following chronic alcohol intake and an acute alcohol withdrawal period (24 h), there was increased total spine density in the NAc in both sexes, an effect driven by increased thin spines. In fact, these spine changes positively correlated with alcohol preference during the final week of drinking. Chronic ketamine intake and a prolonged abstinence from this drug (3 weeks), however, led to reduced thin and increased mushroom spines in the NAc in both sexes, and we showed that the total number of infusions during self-administration positively correlated with increased mushroom type spines. Importantly, high-alcohol intake male rats did not display increased mushroom spines. It is possible that this was the result of lower ketamine intake when compared with other groups. Alternatively, low- and high-alcohol intake female rats not only displayed increased thin spines but also increased mushroom spines, suggesting that these differences in plasticity in spines may mediate differences in interactions between alcohol and ketamine between sexes.

In conclusion, we demonstrated a clear relationship between ketamine and alcohol in behavior and brain morphology, and our data suggest that that ketamine could be a viable treatment for AUD in male but not female subjects. Still, studies have shown that interactions between alcohol and ketamine are associated with enhanced apoptosis in brain regions such as the prefrontal cortex and hippocampus along with behavioral phenotypes of depression-like behavior (Zuo et al., 2017; Li et al., 2019). Therefore, more work is necessary in both sexes to better understand the interactions between ketamine and alcohol and the use of ketamine for treating AUD. Of course, clinical studies are needed to verify our findings in rats and translate them to AUD patients.

## Synthesis

Reviewing Editor: Margaret McCarthy, Univ of Maryland School of Medicine Decisions are customarily a result of the Reviewing Editor and the peer reviewers coming together and discussing their recommendations until a consensus is reached. When revisions are invited, a fact-based synthesis statement explaining their decision and outlining what is needed to prepare a revision will be listed below. The following reviewer(s) agreed to reveal their identity: Jun-Xu Li. Note: If this manuscript was transferred from JNeurosci and a decision was made to accept the manuscript without peer review, a brief statement to this effect will instead be what is listed below.

In this revised manuscript, the authors have addressed many of the prior review concerns. However, not all of the noted issues have been fully resolved as follows:

1. Statistical concerns remain with the same data sets being analyzed more than once in separate analyses.

2. Many of the conclusions are focused on differences in responding between males and females. However, these data are presented on separate graphs. If the intent of the analyses is to probe for such sex differences, the data should be presented on the same graph to allow for proper evaluation of the data among groups.

3. In several cases, statistical results are provided in the absence of any data (‘data not shown’). In the very least, the mean and SEM should be provided within the results text to allow for evaluation of the data.

4. Data are indicated as not being shown for the differences in alcohol intake and preference (results paragraph 1). However, aren't these data presented in Figure 1b and 1c? If so, why aren't there any denoted statistical differences between the groups on the graphs?

5. The conversion of the PR data to log values is not the convention in the addiction field for such data sets. Analyzing the data in this way appears to be a manipulation to provide significant differences between the groups and makes the data difficult to properly interpret. For instance, what is the significance of a ‘1’ difference in the Log2Breakpoint between the groups? Is this a ‘big’ difference in motivation, or a ‘small’ difference? If variability is noted with the PR values, perhaps it would be of benefit to include the individual data points for each subject on the graphs to demonstrate this variability.

6. Furthermore, the PR differences are not maintained across all sessions, but the authors have overlooked this discrepancy within the data. It would be of benefit to at least address this in the discussion.

7. A significant portion of the conclusions are based on ‘statistical trends’. If the differences are not statistically significant, they are not different and should not be presented as such. Perhaps it would be of benefit to increase the number of subjects per group to allow for sufficient power to detect lower effect sizes with the statistical analyses.

8. The acronym IA2BC20% needs to be fully defined within the methods. It is unclear as to what this specifically means regarding the procedure.

9. The X axis labeling in Figure 1 is unclear. It appears that there were only 8 consecutive sessions, but this is not valid since other sessions were conducted in between 6 and 7. Perhaps it would be clearer to indicate ‘reinstatement 1 and 2’ (or R1, R2), as opposed to ‘7’ and ‘8’.

Minor

1. The rationale for the studies repeatedly notes that these studies seek to examine ‘safety of such repeated treatments’ for ketamine. However, safety is not directly assessed in the studies outlined. Along these lines, is is indicated that a major safety concern is noted due to ketamine's effects on NAc plasticity - this conjecture is confusing as it is unclear as to how a change in plasticity would be a ‘safety’ issue.

2. The t statistic reporting needs to be properly shown with degrees of freedom. This is inconsistent throughout the paper.
